# Distinctive Roles of Canonical and Noncanonical Wnt Signaling in Human Embryonic Cardiomyocyte Development

**DOI:** 10.1016/j.stemcr.2016.08.008

**Published:** 2016-09-15

**Authors:** Silvia Mazzotta, Carlos Neves, Rory J. Bonner, Andreia S. Bernardo, Kevin Docherty, Stefan Hoppler

**Affiliations:** 1 Institute of Medical Sciences, University of Aberdeen, Aberdeen AB25 2ZD, UK; 2 The Francis Crick Institute, Mill Hill Laboratory, London NW7 1AA, UK; 3 Anne McLaren Laboratory for Regenerative Medicine, University of Cambridge, West Forvie Building, Robinson Way, Cambridge CB2 0SZ, UK

## Abstract

Wnt signaling is a key regulator of vertebrate heart development; however, specific roles for human cardiomyocyte development remain uncertain. Here we use human embryonic stem cells (hESCs) to analyze systematically in human cardiomyocyte development the expression of endogenous Wnt signaling components, monitor pathway activity, and dissect stage-specific requirements for canonical and noncanonical Wnt signaling mechanisms using small-molecule inhibitors. Our analysis suggests that WNT3 and WNT8A, via FZD7 and canonical signaling, regulate *BRACHYURY* expression and mesoderm induction; that WNT5A/5B, via ROR2 and noncanonical signaling, regulate *MESP1* expression and cardiovascular development; and that later in development WNT2, WNT5A/5B, and WNT11, via FZD4 and FZD6, regulate functional cardiomyocyte differentiation via noncanonical Wnt signaling. Our findings confirm in human development previously proposed roles for canonical Wnt signaling in sequential stages of vertebrate cardiomyogenesis, and identify more precise roles for noncanonical signaling and for individual Wnt signal and Wnt receptor genes in human cardiomyocyte development.

## Highlights

• hESCs were used to study Wnt signaling during human cardiomyocyte development
• Previously proposed roles for canonical Wnt signaling were confirmed in human
• Specific roles for noncanonical Wnt signaling were identified in cardiomyogenesis
• Individual Wnt signal and receptor genes were identified in human cardiomyogenesis

## Graphical Abstract

**Figure:**
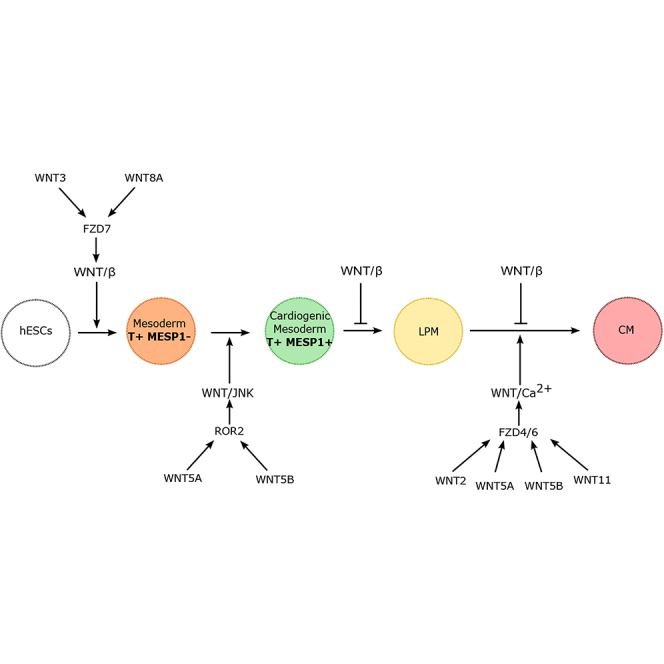

## Teaser

Wnt signaling is an established key regulator of vertebrate heart development, but its role in human cardiomyocyte development remains unclear. Here, Hoppler and colleagues use hESCs to study canonical and noncanonical Wnt pathway activity and identify Wnt signal and receptor genes during human cardiomyocyte development. They identify roles for Wnt signaling that are relevant for congenital defects studies and regenerative medicine applications.

## Introduction

The human heart develops as the first functional organ in the embryo. In the lateral plate mesoderm (LPM) cardiac progenitor cells are induced, which can subsequently differentiate into heart muscle cells (cardiomyocytes) (Rosenthal and Harvey, 2010, Sylva et al., 2014). Wnt signaling, a well-known key regulator of vertebrate cardiomyocyte differentiation (Hoppler et al., 2014), acts through several molecular mechanisms (Hoppler and Nakamura, 2014): a β-catenin-dependent, so-called canonical pathway, and β-catenin-independent, so-called noncanonical pathways, among which a JNK-dependent pathway is prominent during heart development (Gessert and Kühl, 2010, Gessert et al., 2008, Pandur et al., 2002a, Pandur et al., 2002b).

Several studies in mouse and other experimental models have described diverse, and often opposing, effects of canonical and noncanonical Wnt signaling on subsequent cardiomyocyte differentiation, leading to the argument that particularly the JNK-mediated noncanonical pathway may function in this context to antagonize canonical Wnt signaling (Abdul-Ghani et al., 2011, Cohen et al., 2012; reviewed by Hoppler et al., 2014). In addition, canonical Wnt signaling has been shown to play multiple and conflicting roles at different stages of heart development (Gessert and Kühl, 2010, Naito et al., 2006). However, specific roles for Wnt signaling in human cardiomyocyte development remain unclear, particularly regarding which endogenous Wnt ligands and Wnt receptors are involved.

In this study we therefore used human embryonic stem cells (hESCs) to dissect stage-specific requirements of Wnt signaling and to identify endogenous Wnt ligand and receptor genes directing human embryonic cardiomyocyte development.

## Results

### Investigating Wnt Signaling during Human Cardiomyocyte Development Using hESCs

**Figure 1:**
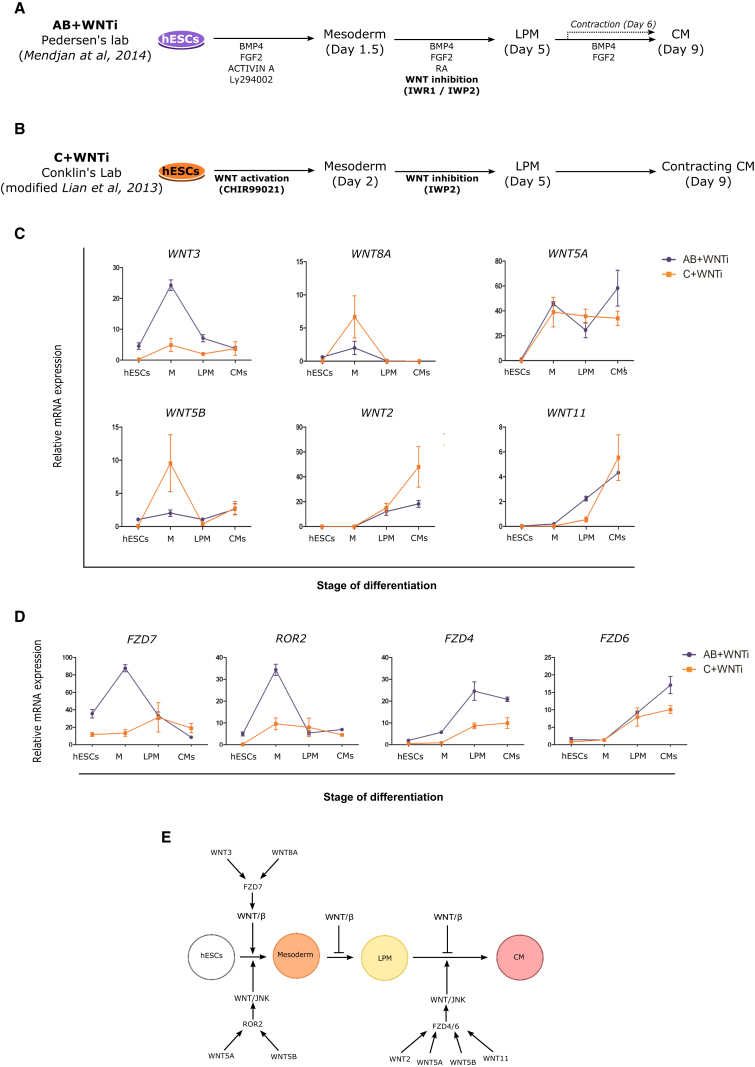
*WNT* Signal and Receptor Gene Expression in Human Cardiomyocyte Development Using hESC Differentiation Protocols (A) Schematic description of the cardiomyocyte differentiation protocol AB + WNTi. This established protocol guides pluripotent hESC first through mesoderm (M) induction with reagents including activin A and BMP4, AB, and then drives lateral plate mesoderm (LPM) and ultimately cardiomyocyte (CM) development with reagents including Wnt inhibitors, WNTi (IWR1 or IWP2). (B) Schematic description of the cardiomyocyte differentiation protocol C + WNTi. This established protocol drives mesoderm induction by adding the Wnt signaling agonist CHIR99021, C, and then promotes cardiomyocyte development with Wnt signaling inhibitors, WNTi (i.e., IWP2). (C) *WNT* gene expression in human cardiomyocyte differentiation protocols by qPCR analysis. Note the prominent expression of *WNT3*, *8A*, *5A*, and *5B* early during mesoderm induction and expression of *WNT5A*, *5B*, *2*, and *11* later during cardiomyocyte differentiation. (D) *FZD* receptor gene and WNT co-receptor gene expression in human cardiomyocyte differentiation protocols analyzed by qPCR. Note the prominent expression of *FZD7* and *ROR2* early during mesoderm induction and *FZD4* and *FZD6* later during cardiomyocyte differentiation. (E) Schematic representation of the working hypothesis about Wnt signaling mechanisms regulating human cardiomyocyte development early during mesoderm induction and later during cardiomyocyte differentiation. qPCR data are presented as means ± SEM for n = 3 independent experiments. *GAPDH* was used as a housekeeping gene. See Experimental Procedures for further details of cardiomyocyte differentiation protocols. See Figure S2 for comprehensive expression analysis of all *WNT* genes, all *FZD* genes, and several WNT co-receptor genes, and Table S1 for information on spatial expression of mouse homologs.

To study sequential stages of human cardiomyocyte development in vitro, we explored established hESC differentiation protocols. With two hESC cell lines (H9 and H7) we used an activin/bone morphogenetic protein (BMP)-based protocol, hereby named the AB + WNTi protocol (Figure 1A and Experimental Procedures, modified from Mendjan et al., 2014), and a CHIR99021-based protocol, hereby named C + WNTi (Figure 1B and Experimental Procedures, modified from Lian et al., 2013). As previously shown, these protocols lead to loss of pluripotency, induction of mesoderm identity, development of cardiac precursor identity in an LPM context, and differentiation into beating primary cardiomyocytes (Figures S1C–S1F). These protocols are thereby clearly following the normal developmental progress in the embryonic heart (Rosenthal and Harvey, 2010, Sylva et al., 2014). Beating activity is observed as early as day 6 for the AB + WNTi protocol (Movie S1) and day 9 for the C + WNTi protocol (Movie S2).

Consistent with findings from model systems (e.g., Singh et al., 2007, Ueno et al., 2007), inhibition of canonical Wnt signaling following mesoderm induction is essential for efficient subsequent human cardiomyocyte differentiation (Figures S1A and S1B) (see also Burridge et al., 2014, Cao et al., 2013, Laflamme et al., 2007, Lian et al., 2012, Mendjan et al., 2014, Paige et al., 2010, Willems et al., 2011). In this study we mainly use the small-molecule Wnt inhibitor IWP2 (an inhibitor of Wnt ligand secretion which, as such, interferes with any Wnt-mediated signaling, irrespective of pathway mechanisms) and also IWR1 (a specific inhibitor of canonical Wnt/β-catenin signaling) (Chen et al., 2009). Either inhibitor promotes subsequent cardiomyocyte differentiation, confirming that inhibition of canonical Wnt signaling is sufficient in this context for differentiation of hESCs into functional cardiomyocytes (Figure S1).

### Endogenous Expression of Wnt Signaling Ligands and Receptors Suggests Stage-Specific Roles for Wnt Signaling in Cardiomyocyte Differentiation

Manipulation of Wnt signaling is a well-established experimental tool for driving cardiomyocyte differentiation in hESC protocols (e.g., Lian et al., 2012), suggesting a key role for Wnt pathways in endogenous cardiomyocyte development. So far, information about specific Wnt signals and receptors is only available from animal models (Table S1; Eisenberg and Eisenberg, 1999, Hardy et al., 2008, Kilian et al., 2003, Lavery et al., 2008, Pandur et al., 2002a, Ulrich et al., 2005). To identify WNT signals and receptors that may regulate cardiomyocyte development in humans, we systematically studied the expression of all 19 genes encoding WNT signals (Figure S2A), of all ten frizzled (FZD) receptors, and of WNT co-receptors at key stages of human cardiomyocyte development (Figure S2B), using the aforementioned protocols.

We discovered that *WNT3* and *WNT5A* are particularly highly expressed during mesoderm induction, together with *WNT8A* and *WNT5B*. Much later in development, during cardiomyocyte differentiation, high expression of *WNT2*, *WNT5A*, *WNT5B*, and *WNT11* was observed (Figures 1C and S2A). We also uncovered strong expression of both *FZD7* and *ROR2* during mesoderm induction, and *FZD4* and *FZD6* expression during cardiomyocyte differentiation (Figures 1D and S2B). *WNT3* and *WNT8A* are known to activate the canonical Wnt pathway (Yamaguchi, 2008) while *WNT5A*, *WNT5B*, and *WNT11* are often associated with noncanonical Wnt signaling mechanisms (Cohen et al., 2012, He et al., 1997, Pandur et al., 2002a, Yamaguchi, 2008).

These results allowed us to formulate an initial working hypothesis (Figure 1E) proposing that canonical Wnt signaling is activated during mesoderm induction by WNT3 and WNT8A, via FZD7; and that JNK-mediated noncanonical Wnt signaling is activated by WNT5A and WNT5B, probably involving ROR2. During cardiomyocyte differentiation stages, the expression of *WNT5A*, *WNT5B*, and *WNT11* suggests that these ligands activate noncanonical Wnt signaling via *FZD4* and *FZD6*, and possibly together with *WNT2*, presumably through a JNK-mediated mechanism as previously suggested in the mouse system (Gessert and Kühl, 2010, Pandur et al., 2002a). These proposed functions of human genes in this working hypothesis are on the whole consistent with the expression of the mouse homologs, where such information is available (Table S1). However, we proceeded to test further predictions of this working hypothesis.

### Canonical and Noncanonical Wnt Pathway Activation during Different Stages of Human Cardiomyocyte Differentiation

To test our working hypothesis (Figure 1E) and further explore this model, we studied Wnt signaling pathway activity during sequential stages of the cardiomyocyte differentiation protocols.

Canonical Wnt signaling activity was monitored by following the expression levels of the known Wnt/β-catenin target gene *AXIN2* (Yan et al., 2001) and by analyzing protein abundance and localization of active β-catenin (ABC) (Staal et al., 2002). Our molecular understanding of noncanonical WNT signaling is still rudimentary, and methods for monitoring noncanonical Wnt signaling activity are therefore less well established; specifically, there is no reliable pathway indicator gene for noncanonical WNT signaling. Since a JNK-dependent Wnt pathway is prominent during heart development (Gessert and Kühl, 2010, Hoppler et al., 2014), we monitored expression of the recently identified target gene of noncanonical Wnt/JNK signaling, *ALCAM* (Choudhry and Trede, 2013, Cizelsky et al., 2014), and protein abundance and cellular localization of phosphorylated JNK (Pandur et al., 2002a).

**Figure 2:**
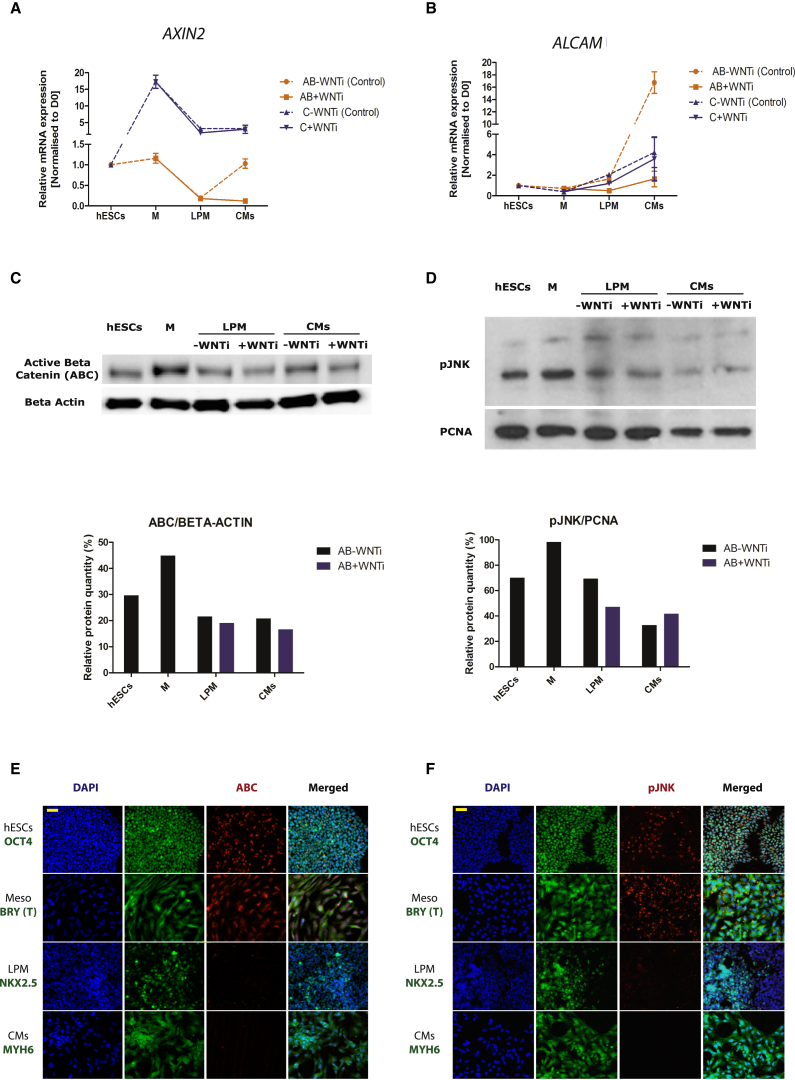
Activity of Wnt Signaling Pathways in Human Cardiomyocyte Differentiation Protocols (A) Gene expression (analyzed by qPCR) of the canonical Wnt target gene *AXIN2*. Note *AXIN2* expression confined to mesoderm induction and reduced expression during cardiomyocyte differentiation. (B) Gene expression (analyzed by qPCR) of the proposed noncanonical Wnt target gene *ALCAM*. Note *ALCAM* expression only increasing at later stages of the protocol, but decreasing following addition of Wnt inhibitor. (C) Top panel: western blot analysis of active β-catenin (ABC) and β-actin in the AB −/+ WNTi (i.e., IWP2) protocols. Bottom panel: quantified western analysis showing protein abundance of ABC relative to the housekeeping protein β-actin. Note increased ABC abundance confined to mesoderm induction. (D) Top panel: western blot analysis of phosphorylated JNK (pJNK) and proliferating cell nuclear antigen (PCNA) in the AB −/+ WNTi (i.e., IWP2) protocols. Bottom panel: quantified western analysis showing protein abundance of pJNK relative to the housekeeping protein PCNA. Note increased pJNK abundance mostly confined to mesoderm induction. (E) Tissue-wide analysis of Wnt/β-catenin pathway activity. Immunocytochemistry analysis of activated β-catenin (ABC) at different stages of cardiomyocyte differentiation protocols, marked by the localization of OCT4 (pluripotent hESCs), BRY (T) (mesoderm), NKX2.5 (LPM), and MYH6 (cardiomyocytes). Note uniform nuclear localization of ABC confined to mesoderm induction stages. Scale bar, 50 μm. (F) Tissue-wide analysis of Wnt/JNK pathway activity. Immunocytochemistry analysis of phosphorylated JNK (pJNK) at different stages of cardiomyocyte differentiation protocols, marked by the localization of OCT4 (pluripotent hESCs), BRY (T) (mesoderm), NKX2.5 (LPM), and MYH6 (cardiomyocytes). Note uniform cytoplasmic pJNK localization early during mesoderm induction. Scale bar, 50 μm. qPCR data are presented as means ± SEM normalized to day 0 for n = 3 independent experiments. *GAPDH* was used as a housekeeping gene.

As predicted by the working hypothesis (Figure 1E), we find that *AXIN2* expression is high during mesoderm induction (Figure 2A), and consistently the ABC protein is enriched (Figure 2C) and has a nuclear localization in induced mesoderm (Figure 2E), demonstrating active canonical Wnt signaling during these early stages. Contrarily, *AXIN2* expression levels clearly reveal that canonical Wnt signaling is reduced after mesoderm induction in these human cardiomyocyte differentiation protocols, and remains reduced during cardiomyocyte differentiation (Figure 2A). These findings are further supported by the low levels of ABC protein at these later stages (Figures 2C and 2E).

Consistent with the working hypothesis (Figure 1E), we find activation of JNK-mediated signaling during mesoderm induction when analyzing phosphorylated JNK protein levels and cellular localization (Figures 2D and 2F), and *ALCAM* expression gradually increasing at later stages (Figure 2B). However, *ALCAM* is not expressed during mesoderm induction and might therefore not be a good indicator of noncanonical Wnt/JNK signaling at this stage. At later stages analysis of phosphorylated JNK did not consistently indicate an increase, which would be contrary to the prediction of the working hypothesis and the ALCAM expression data. However, as expected, addition of IWP2 reduces *ALCAM* expression and JNK phosphorylation.

Altogether, these data support our working hypothesis (Figure 1E), as far as showing that canonical Wnt signaling is confined to mesoderm induction, and suggest potential noncanonical Wnt signaling activity during mesoderm induction and cardiomyocyte differentiation. We therefore proceeded to further test our working hypothesis by experimentally interfering with the Wnt pathway at critical stages of cardiomyocyte development.

### Both β-Catenin- and JNK-Mediated Signaling Are Required Early during Mesoderm Development

**Figure 3:**
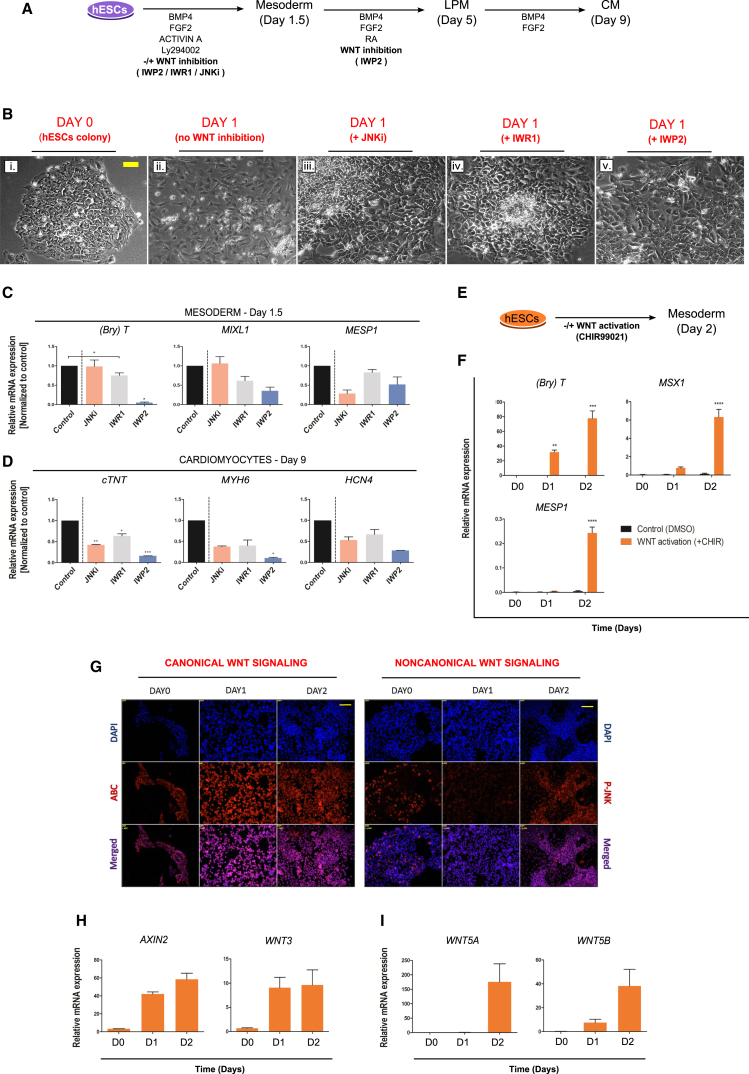
Wnt Signaling Function during Mesoderm Development (A) Schematic description of the cardiomyocyte differentiation protocol to study the requirement for Wnt signaling pathway function during mesoderm development. The protocol AB + WNTi (as in Figure 1A) is modified to inhibit Wnt signaling mechanisms (by addition of IWP2, IWR1, or JNKi [SP600125]) during mesoderm induction. (B) Morphology of cultured cells (bright-field micrographs; scale bar, 400 μm) at the start of the differentiation protocol (day 0, panel Bi) and after 1 day (day 1, panels Bii–Bv) under different experimental conditions as shown in (A). (Bii) represents the control (no WNT inhibition); (Biii) represents cells treated with JNKi; (Biv) represents cells treated with IWR1; (Bv) represents cells treated with IWP2. (C) Gene expression (qPCR analysis, relative to control) of markers of mesoderm induction at day 1.5 in different conditions as shown in (A). Note that JNK signaling appears to be specifically required for *MESP1* expression. (D) Gene expression (qPCR analysis, relative to control) of cardiomyocyte markers at day 9 in the different conditions shown in (A). (E) Schematic description of the protocol to study sufficiency in this context of Wnt signaling pathway function for mesoderm development (as in Figure 1B). (F) Gene expression of mesoderm markers (qPCR analysis) on day 0, day 1, and day 2 in the different conditions shown in (E). Note that *MESP1* expression is induced later than *BRY* (*T*) and *MSX1*. (G) Activity of Wnt signaling mechanisms during mesoderm development, in the presence of canonical Wnt activation (CHIR99021) as shown in (E). Immunocytochemistry of activated β-catenin (ABC) and phosphorylated JNK (pJNK) suggests canonical pathway activity already on day 1, but noncanonical pathway activity mainly on day 2 during mesoderm development. Scale bar, 100 μm. (H) qPCR analysis showing expression of the canonical Wnt target gene *AXIN2* and of *WNT3* already on day 1, in the presence of canonical Wnt activation (CHIR99021) as shown in (E). (I) qPCR analysis showing expression of *WNT5A* and *5B* mainly on day 2 during mesoderm development, in the presence of canonical Wnt activation (CHIR99021) as shown in (E). qPCR data are presented as means ± SEM for n = 3 independent experiments. *GAPDH* was used as a housekeeping gene. Asterisk indicates a statistically significant difference between experimental and control samples detected using one-way ANOVA (^∗^p < 0.05) for (C) and (D) and two-way ANOVA for (F). ^∗^p < 0.05; ^∗∗^p < 0.01; ^∗∗∗^p < 0.001; ^∗∗∗∗^p < 0.0001.

To investigate any requirement for canonical and noncanonical Wnt signaling during mesoderm induction, we manipulated the AB + WNTi protocol by adding at this stage the small molecules IWP2, IWR1, or JNKi (Figure 3A). IWP2 is used as above to interfere with Wnt signaling in general, and IWR1 as a specific inhibitor of canonical Wnt/β-catenin signaling (Chen et al., 2009). JNKi (SP600125) is used to inhibit JNK-dependent noncanonical Wnt signaling (Pandur et al., 2002a).

Mesoderm induction is characterized by a dramatic change of cell morphology, from round and small hESCs tightly organized in colonies, to fibroblastoid mesodermal cells that are well spread out from the original colony. Experimental inhibition of either Wnt pathway clearly interfered with these morphological rearrangements (Figure 3B) and, therefore, mesoderm induction.

Expression of the mesodermal markers *BRACHYURY* (*BRY* [*T*]) and *MIXL1* is found to be reduced following inhibition of canonical Wnt signaling in the AB + WNTi protocol, but only the expression of the earliest cardiogenic marker *MESP1* (David et al., 2008) is decreased following inhibition of noncanonical WNT/JNK signaling (Figure 3C). These effects on mesoderm formation lead to expected consequences later, particularly reduced levels of expression of the cardiac markers *cTNT*, *MYH6*, and *HCN4* (Figure 3D). These data suggest that both branches of Wnt signaling are required during initial mesodermal commitment; however, canonical and noncanonical Wnt signaling play different roles at this early stage.

To study any sufficiency for canonical WNT signaling in this context, we used the C + WNTi protocol. Experimental activation of canonical Wnt signaling (Figure 3E) with CHIR99021 leads to expression of *BRY* (*T*) and *MSX1* (starting on experimental day 1) and more delayed *MESP1* (experimental day 2) (Figure 3F). Conversely, absence of exogenous Wnt activation results in lack of mesodermal differentiation. More detailed analysis of Wnt signaling activity at these stages shows that the Wnt/β-catenin pathway is active earlier than the JNK-dependent pathway (experimental day 1 and day 2, respectively) (Figure 3G), which is also supported by *AXIN2* and *WNT3* expression (associated with canonical Wnt signaling, Figure 3H) being earlier than *WNT5A* and *WNT5B* (associated with noncanonical Wnt signaling, Figure 3I). Together, these results show sequential activation and confirm different roles for canonical and noncanonical WNT signaling during human mesoderm induction.

### Canonical and Noncanonical Wnt Signaling Have Different Roles in Late Stages of Cardiomyocyte Differentiation

**Figure 4:**
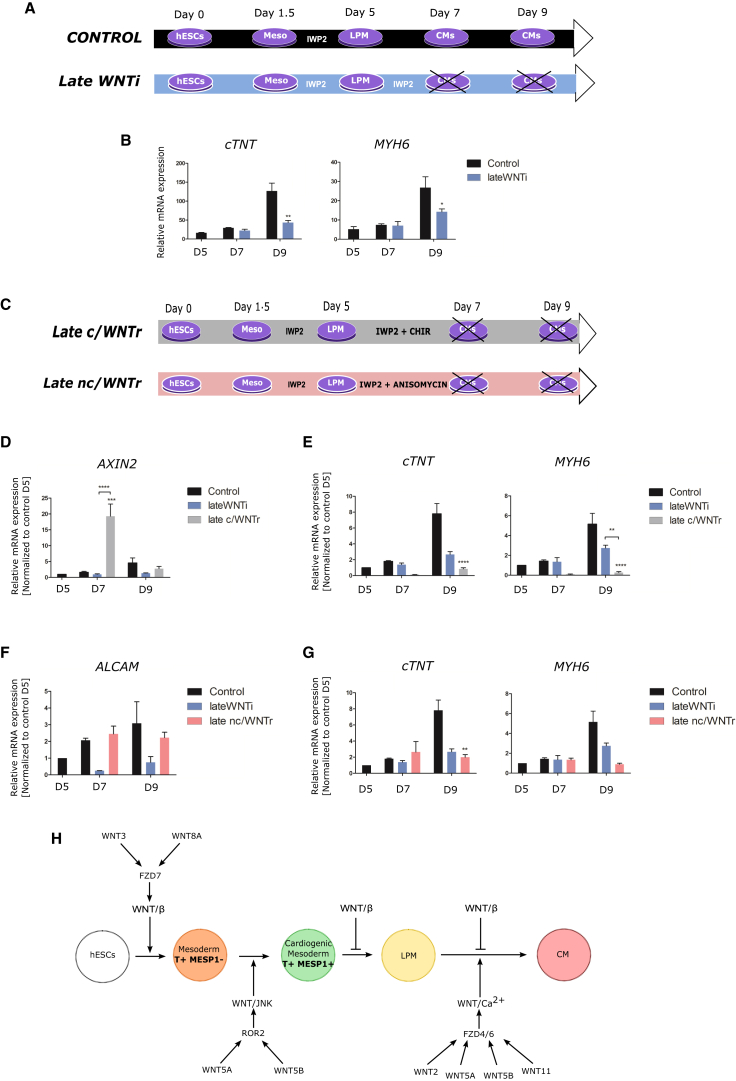
Wnt Signaling Function during Cardiomyocyte Differentiation (A) Schematic description of the protocol to study the requirement for Wnt signaling pathway function during cardiomyocyte differentiation. The protocol AB + WNTi (as in Figure 1A) is modified to extend the inhibition of Wnt signaling mechanisms (by addition of IWP2) until day 7 (Late WNTi). AB + WNTi protocol is used as a control, as indicated. (B) Gene-expression analysis (qPCR) of cardiomyocyte markers with and without extended Wnt inhibition, as in (A). Note the reduced cardiomyocyte marker gene expression with extended Wnt inhibition. (C) Schematic description of the protocol to study whether Wnt signaling requirement can be rescued by reinstating canonical Wnt/β-catenin signaling (with CHIR99021, Late c/WNTr) or noncanonical Wnt/JNK signaling (with anisomycin, Late nc/WNTr). (D) Gene-expression analysis (qPCR) of the canonical Wnt target gene *AXIN2*. Note strong induction of *AXIN2* expression by addition of CHIR99021. (E) Gene-expression analysis (qPCR) of cardiomyocyte markers in the different conditions shown in (C). Note that reinstating canonical Wnt/β-catenin signaling further reduces cardiomyocyte marker gene expression. (F) Gene-expression analysis (qPCR) of the proposed noncanonical Wnt target gene *ALCAM* in the different conditions shown in (C). Note strong induction of *ALCAM* expression by addition of anisomycin. (G) Gene-expression analysis (qPCR) of cardiomyocyte markers in the different conditions shown in (C). Note that reinstating noncanonical Wnt/JNK signaling fails to rescue cardiomyocyte marker gene expression. (H) Schematic description of conclusions. Note that the function of canonical and noncanonical Wnt signaling early during mesoderm development can be separated temporally and in terms of marker gene induction. Further note that later canonical Wnt signaling is still inhibitory, while noncanonical Wnt signaling is required for cardiomyocyte differentiation (see text for further details). qPCR data are presented as means ± SEM for n = 3 independent experiments. *GAPDH* was used as a housekeeping gene. Asterisk indicates a statistically significant difference between samples as indicated, which was detected using two-way ANOVA. ^∗^p < 0.05; ^∗∗^p < 0.01; ^∗∗∗^p < 0.001; ^∗∗∗∗^p < 0.0001.

Hardly anything is currently known about canonical and noncanonical Wnt signaling at later stages of human cardiomyocyte differentiation. We therefore studied molecular differentiation of cardiomyocytes when inhibition of Wnt signaling by IWP2 was experimentally prolonged (Figure 4A), and found decreased expression of cardiomyocyte differentiation markers (Figure 4B), suggesting that Wnt signaling function is required at these stages for cardiomyocyte differentiation.

We now wondered whether we could rescue cardiomyocyte differentiation by experimentally reinstating β-catenin or JNK pathway activity (Figure 4C). Activation of β-catenin signaling boosted *AXIN2* expression (Figure 4D), as expected, but caused further decreased cardiomyocyte differentiation marker expression (Figure 4E), confirming that canonical Wnt signaling at these stages is incompatible with cardiomyocyte differentiation. Activation of JNK signaling boosted *ALCAM* expression (Figure 4F), as expected, but unexpectedly did not rescue the loss of differentiation induced by IWP2 (Figure 4G).

## Discussion

Vertebrate and invertebrate models have proved fundamental for gaining an understanding of animal heart development and the signaling and transcriptional network mechanisms governing this process. However, the molecular mechanisms underlying human heart development are still largely unclear. hESCs offer unprecedented opportunities to model and study human heart development in vitro. However, so far most of the effort in the field has been directed toward designing efficient protocols to differentiate human cardiomyocytes in vitro. Interestingly, a vast majority of these protocols relies on experimental manipulation of Wnt signaling mechanisms (e.g., Bauwens et al., 2011, Burridge et al., 2014, Chen et al., 2012, Elliott et al., 2011, Gonzalez et al., 2011, Hemmi et al., 2014, Karakikes et al., 2014, Kattman et al., 2011, Kempf et al., 2014, Lian et al., 2012, Otsuji et al., 2014, Paige et al., 2010, Phillips et al., 2008, Ting et al., 2014, Yang et al., 2008, Zhang et al., 2015), suggesting important roles for WNT signaling also in human heart development. However, while Wnt signaling clearly represents a key regulator of vertebrate heart development and particularly of cardiomyocyte differentiation (reviewed by Hoppler et al., 2014), at present there is no detailed understanding of the players and the fundamental roles of Wnt signaling at sequential developmental stages leading to human embryonic cardiomyocyte differentiation. We therefore specifically set out to test whether knowledge from animal model systems would be confirmed for human cardiomyocyte development. Using established hESC differentiation protocols, we studied the activity and requirement of Wnt signaling pathways and identified *WNT* signal and receptor genes during human cardiomyocyte differentiation.

Evidence from model systems had previously suggested a requirement for Wnt signaling during mesoderm induction. In fact, Brachyury is a known target of β-catenin-mediated Wnt signaling (e.g., Arnold et al., 2000, Mendjan et al., 2014, Yamaguchi et al., 1999, Zhang et al., 2013), while WNT/JNK signaling had previously been associated with morphogenesis at gastrulation (Hardy et al., 2008, Tada et al., 2002, Tada and Kai, 2009). Here, we not only confirm that similarly hESCs can only efficiently differentiate in vitro into mesoderm when Wnt signaling is active, but also show that canonical Wnt signaling acts before noncanonical Wnt signaling in humans (Figure 4H). Our data suggest that WNT3 and WNT8A regulate *BRY* (*T*) expression and mesoderm induction via the canonical pathway, after which WNT5A and WNT5B activate JNK-mediated pathway activity to regulate *MESP1* expression, thereby indicating a role for WNT/JNK signaling that goes beyond regulating morphogenesis during gastrulation in the intact embryo. Consistently, our results also show expression of *FZD7* and the noncanonical receptor *ROR2* during mesoderm induction. Interestingly *FZD7* had previously been identified in pluripotent hESCs, where it plays a role in canonical WNT3-mediated self-renewal (Fernandez et al., 2014). Our results suggest that WNT3 and WNT8A and the canonical pathway regulate mesoderm induction via the FZD7 receptor (although additional roles for FZD7 in mediating noncanonical mechanisms cannot be ruled out, e.g., Medina et al., 2000), while ROR2 mediates WNT5A/B role in the commitment of the earliest cardiogenic mesoderm (i.e., MESP1-positive cells).

We further confirm in human cells that inhibition of specifically canonical Wnt signaling following mesoderm induction is both necessary and sufficient for subsequent efficient cardiomyocyte differentiation (Figure 4H). Experimental activation of noncanonical Wnt/JNK signaling has been shown in a variety of experimental models to promote cardiac specification (Bisson et al., 2015, Eisenberg and Eisenberg, 1999, Terami et al., 2004), which, it has been argued, may function by antagonizing canonical pathway activity (reviewed in Hoppler et al., 2014). However, we observe little activation of noncanonical Wnt/JNK signaling following mesoderm induction and little evidence of pathway-relevant Wnt ligand or Wnt receptor gene expression, which suggests that noncanonical Wnt/JNK signaling may not be required at this specific stage. Consistently, we find that experimental inhibition of JNK signaling alone at this stage does not affect differentiation of mesodermal cells into LPM and subsequently into cardiomyocytes. The effects observed by others on cardiac specification after experimental activation of noncanonical Wnt/JNK signaling may be related to the earlier function of Wnt/JNK signaling we have discovered (see above).

Finally, until now nothing was known about the role of Wnt signaling during differentiation from cardiac precursors into human cardiomyocytes. In animal model systems, a prominent role of noncanonical Wnt/JNK signaling at this stage has been suggested, while it is still not clear whether canonical Wnt signaling may play further roles following cardiac mesoderm induction for subsequent cardiomyocyte differentiation (both reviewed in Gessert and Kühl, 2010). We observe sustained expression of presumably noncanonical *WNT5A*, *WNT5B*, and *WNT11* at this stage (Figure 4H), but also *WNT2*, which was conventionally believed to activate β-catenin-mediated signaling (Goss et al., 2009). When Wnt signaling was inhibited at this late stage, cardiomyocyte differentiation was negatively affected. However, activation of canonical Wnt signaling at this stage was found to be detrimental for terminal cardiomyocyte differentiation, suggesting a role solely for noncanonical Wnt signaling during cardiomyocyte differentiation. The involvement of *wnt2* in cardiomyocyte differentiation from ESCs mediated by noncanonical Wnt signaling had previously been identified in the mouse (Onizuka et al., 2012), which our results now suggest are conserved in human development. Our results further suggest that noncanonical signaling at this stage is mediated by *FZD4* (Abdul-Ghani et al., 2011, Descamps et al., 2012) and *FZD6* (Gray et al., 2011, Guo et al., 2004) receptors. However, experimental activation of JNK signaling alone proved insufficient for rescuing cardiomyocyte differentiation. Therefore, future experiments will be required to explore the role of other noncanonical Wnt signaling pathways in late cardiomyocyte differentiation, which may work together with or instead of the JNK-mediated pathway. Given that Ca^2+^ is needed for proper cardiac development and heart function, the Wnt/Ca^2+^ pathway may represent a mediator of noncanonical Wnt signaling in this context (Panakova et al., 2010).

Overall, our study demonstrates that canonical and noncanonical Wnt pathway mechanisms have specific roles in regulating human cardiomyocyte differentiation that go beyond simple mutual antagonism. The established hESC differentiation protocols we used clearly proved useful to distinguish different phases of Wnt pathway activity and requirement, as well as stage-specific Wnt signal and Wnt receptor gene expression (Figure 4H). Future analysis may be able to determine more precisely whether canonical and noncanonical Wnt signaling mechanisms operate at exactly the same stage and in exactly the same tissue, and whether specific Wnt ligands and receptors mediate canonical and others noncanonical pathway activation. Given the outstanding impact of cardiovascular disease on society, a more detailed understanding of the regulatory mechanisms underlying human embryonic cardiac muscle commitment and differentiation will provide fundamental insights into the etiology of congenital heart defects, and may also suggest innovative therapeutic applications in the field of regenerative medicine.

## Experimental Procedures

### hESC Differentiation Protocols

#### AB + WNTi

hESCs (NSCB no. WA09 [H9] and WA07 [H7]) were grown on inactivated mouse embryonic fibroblasts, as previously described (Thomson et al., 1998). All experiments were repeated with both cell lines with comparable results, but results presented here are from H9 culture. Differentiation toward mesoderm, LPM, and beating cardiomyocytes was performed in chemically defined medium containing polyvinyl alcohol, essentially as previously described (Bernardo et al., 2011, Mendjan et al., 2014) with only minor modifications (Figure 1A): for the first 36 hr of the protocol cells were stimulated with 20 ng/mL fibroblast growth factor 2 (FGF2),10 μM phosphoinositide 3-kinase inhibitor (LY294002), 20 ng/mL activin A, and 10 ng/mL BMP4, for mesoderm induction; addition of 20 ng/mL FGF2, 50 ng/mL BMP4, 0.5 μM retinoic acid, and 5 μM of Wnt inhibitor (either IWR1 or IWP2) from day 1.5 to day 5 allowed LPM differentiation; finally, treatment with 5 ng/mL FGF2 and 10 ng/mL BMP4 allowed differentiation into cardiomyocytes.

#### C + WNTi

hESCs (NSCB no. WA09 [H9] and WA07 [H7]) were grown on mTeSR1/Matrigel Platform as per the manufacturer's instructions. All experiments were repeated with both cell lines with comparable results, but results presented here are from H7 culture. Differentiation toward mesoderm, LPM, and beating cardiomyocytes was performed essentially as previously described (Lian et al., 2013) with only minor modifications (Figure 1B): for the first 48 hr of the protocol cells were stimulated with 12 μM CHIR99021 in RPMI/low insulin-B27 for mesoderm induction; subsequently, addition of the Wnt inhibitor IWP2 (5 μM) from days 2 to 4 in RPMI/low insulin-B27 allowed LPM differentiation; cells were then grown in RPMI/low insulin-B27 for a further 2 days; finally, medium was changed to RPMI/B27 on day 6 and cells were kept in culture until day 9 to allow differentiation into cardiomyocytes.

## Author Contributions

S.M. carried out the experiments and analysis, helped in experimental design, and wrote the manuscript. C.N. contributed to design, carried out experiments, and advised on analysis and manuscript. R.J.B. carried out experiments. A.S.B. advised on design, methods, and manuscript, and carried out experiments. K.D. advised on design and manuscript. S.H. designed the study and wrote the manuscript.

## Supplemental Information

Document S1. Supplemental Experimental Procedures, Figures S1 and S2, and Tables S1–S3 Document S1. Supplemental Experimental Procedures, Figures S1 and S2, and Tables S1–S3

Movie S1. The AB + WNTi Protocol Leads to Development of Functionally Active Cardiomyocytes Movie S1. The AB + WNTi Protocol Leads to Development of Functionally Active Cardiomyocytes

Movie S2. The C + WNTi Protocol Leads to Development of Functionally Active Cardiomyocytes Movie S2. The C + WNTi Protocol Leads to Development of Functionally Active Cardiomyocytes

Document S2. Article plus Supplemental Information Document S2. Article plus Supplemental Information
