# Supplementary material for: Distinctive Roles of Canonical and Noncanonical Wnt Signaling in Human Embryonic Cardiomyocyte Development
Source: Stem Cell Reports. 2016 Sep 15;7(4):764–76. doi: 10.1016/j.stemcr.2016.08.008 (PMC5063467; doi:10.1016/j.stemcr.2016.08.008)
Supplement: Document S1. Supplemental Experimental Procedures, Figures S1 and S2, and Tables S1–S3 [file mmc1.pdf]

Stem Cell Reports, Volume 7

Supplemental Information

Distinctive Roles of Canonical and Noncanonical Wnt Signaling in Human Embryonic Cardiomyocyte Development

Silvia Mazzotta, Carlos Neves, Rory J. Bonner, Andreia S. Bernardo, Kevin Docherty, and Stefan Hoppler

SUPPLEMENTAL INFORMATION

SUPPLEMENTAL FIGURES

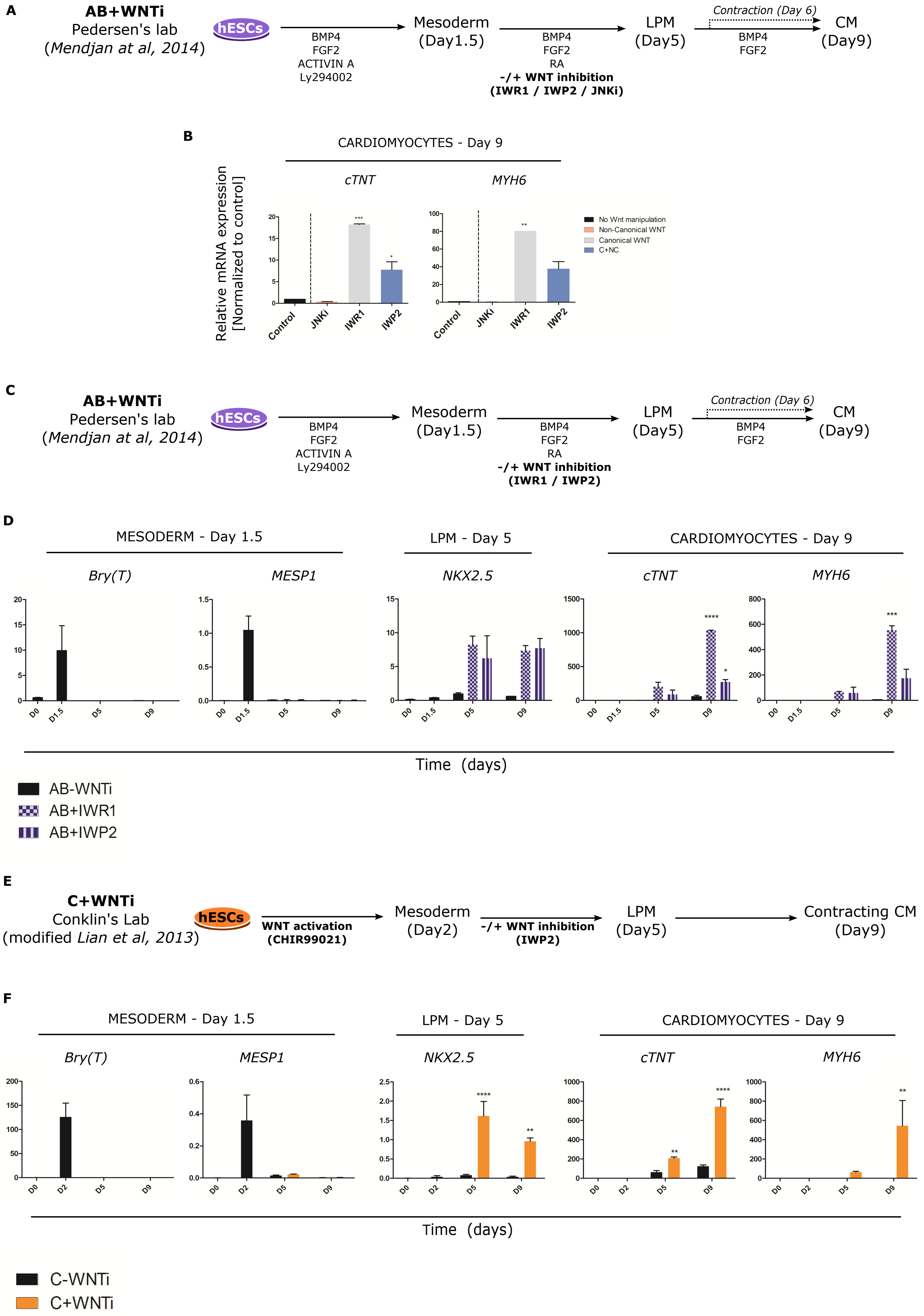

A
AB+WNTi
Pedersen's lab
(Mendjan at al, 2014)
hESCs
BMP4
FGF2
ACTIVIN A
Ly294002
Mesoderm
(Day1.5)
BMP4
FGF2
RA
-/+ WNT inhibition
(IWR1 / IWP2 / JNKi)
LPM
(Day5)
Contraction (Day 6)
BMP4
FGF2
CM
(Day9)
B
CARDIOMYOCYTES - Day 9
cTNT
MYH6
Relative mRNA expression
[Normalized to control]
Control
JNKi
IWR1
IWP2
No Wnt manipulation
Non-Canonical WNT
Canonical WNT
C+NC
C
AB+WNTi
Pedersen's lab
(Mendjan at al, 2014)
hESCs
BMP4
FGF2
ACTIVIN A
Ly294002
Mesoderm
(Day1.5)
BMP4
FGF2
RA
-/+ WNT inhibition
(IWR1 / IWP2)
LPM
(Day5)
Contraction (Day 6)
BMP4
FGF2
CM
(Day9)
D
MESODERM - Day 1.5
LPM - Day 5
CARDIOMYOCYTES - Day 9
Bry(T)
MESP1
NKX2.5
cTNT
MYH6
Relative mRNA expression
D0
D1.5
D5
D9
Time (days)
AB-WNTi
AB+IWR1
AB+IWP2
E
C+WNTi
Conklin's Lab
(modified Lian et al, 2013)
hESCs
WNT activation
(CHIR99021)
Mesoderm
(Day2)
-/+ WNT inhibition
(IWP2)
LPM
(Day5)
Contracting CM
(Day9)
F
MESODERM - Day 1.5
LPM - Day 5
CARDIOMYOCYTES - Day 9
Bry(T)
MESP1
NKX2.5
cTNT
MYH6
Relative mRNA expression
D0
D2
D5
D9
Time (days)
C-WNTi
C+WNTi

Supplemental Figure S1: Study of Wnt signaling requirement for human cardiomyocyte development using hESC differentiation protocols

(**A**) Schematic description of the cardiomyocyte differentiation protocol AB+WNTi, where WNTi is addition of IWP2, IWR1 or JNKi (SP600125) between Day 1.5 and Day 5. (**B**) Cardiomyocyte gene expression study in qPCR on Day 9 of the Protocol described in **S1A**. Note absence of differentiation when just noncanonical Wnt signaling is inhibited. (**C**) Schematic description of the cardiomyocyte differentiation protocol AB+WNTi. (**D**) Gene expression (qPCR analysis) of mesoderm, LPM and Cardiomyocyte markers in the different conditions shown in **Fig. S1C**. Note that inhibition of canonical Wnt signaling is sufficient and necessary for primary cardiomyocyte differentiation. (**E**) Schematic description of the cardiomyocyte differentiation protocol C+WNTi. (**F**) Gene expression (qPCR analysis) of mesoderm, LPM and Cardiomyocyte markers in the different conditions shown in **Fig. S1E.**

qPCR data are presented as mean ± SEM for n=3 biological replicates. *GAPDH* was used as a housekeeping gene. * indicates a statistically significant difference between experimental and control samples detected using 1-way ANOVA for panel B and 2-way ANOVA for panels D and F. * $P<0.05$; ** $P<0.01$; *** $P<0.001$; **** $P<0.0001$.

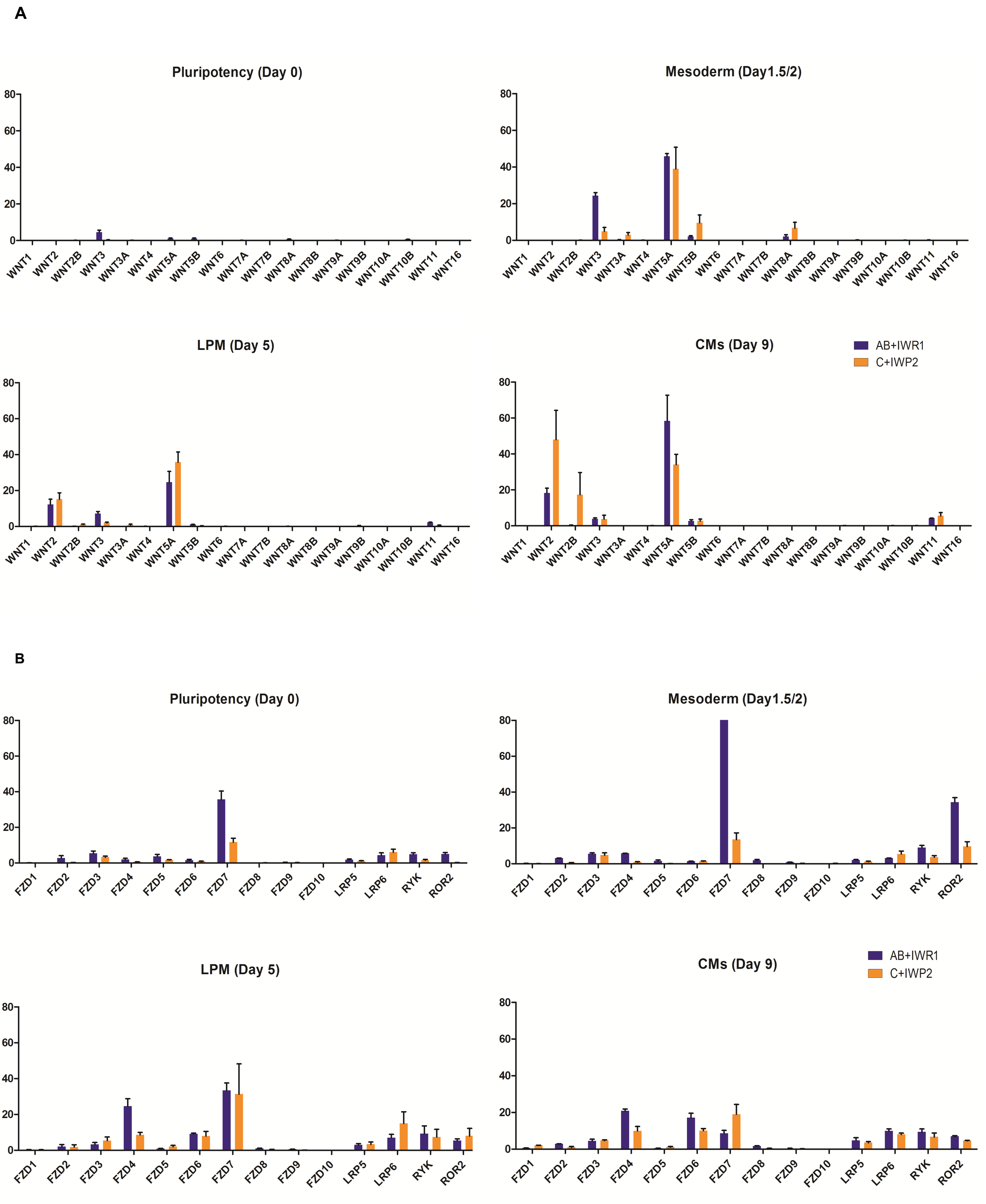
A
Pluripotency (Day 0)
Mesoderm (Day1.5/2)
LPM (Day 5)
CMs (Day 9)
AB+IWR1
C+IWP2
0
20
40
60
80
WNT1
WNT2
WNT2B
WNT3
WNT3A
WNT4
WNT5A
WNT5B
WNT6
WNT7A
WNT7B
WNT8A
WNT8B
WNT9A
WNT9B
WNT10A
WNT10B
WNT11
WNT16
B
Pluripotency (Day 0)
Mesoderm (Day1.5/2)
LPM (Day 5)
CMs (Day 9)
AB+IWR1
C+IWP2
0
20
40
60
80
FZD1
FZD2
FZD3
FZD4
FZD5
FZD6
FZD7
FZD8
FZD9
FZD10
LRP5
LRP6
RYK
ROR2

Supplemental Figure S2: Wnt signal and receptor gene expression in human cardiomyocyte development using hESC differentiation protocols

(**A**) qPCR profile of all Wnt gene expression in AB+WNTi and C+WNTi as indicated. Data is plot by differentiation stage/day. Note prominent expression of *WNT3, 8A, 5A and 5B* early during mesoderm induction and expression of *WNT5A*, *5B*, *2* and *11* later during cardiomyocyte differentiation. (**B**) qPCR profile of all Wnt receptor and co-receptor gene expression in AB+WNTi and C+WNTi as indicated. Data is plot by differentiation stage/day. Note prominent expression of *FZD7* and ROR2 early during mesoderm induction and *FZD4* and *FZD6* later during cardiomyocyte differentiation.

qPCR data are presented as mean ± SEM for n=3 biological replicates. *GAPDH* was used as a housekeeping gene.

SUPPLEMENTAL EXPERIMENTAL PROCEDURES

RNA expression analysis and RT-qPCR

Samples were harvested in Trizol® reagent (Invitrogen). After digestion with DNase I (Invitrogen), 0.5 µg of RNA was used for cDNA synthesis. Quantitative Polymerase Chain Reactions (RT-qPCRs) were then performed using the Sybr Green gene expression assay. Real-time PCR mixtures were prepared following the manufacturer's recommendation (LightCycler® 480 SYBR Green I Master, Roche) for each gene. qPCRs were run in a Roche Lightcycler 480® and normalized to glyceraldehyde 3-phosphate dehydrogenase (GAPDH) in the same run. Data was analyzed using the $2^{-\Delta\Delta CT}$ method (Pfaffl, 2001). Statistical analysis was performed using ANOVA 1way or 2way as appropriate (see figure legends for details), followed by the Bonferroni post hoc test. Primer sequences are available in the supplemental information (**Table S2**).

Western Blot and Protein Quantification

Samples were harvested in Cellytic-M (Sigma). Protein extraction was performed following manufacturer's recommendations. Equal volumes of protein (15µl) were loaded on a gradient 4-12% Bis-Tris NuPage Gel (Life Technologies, USA) and run at 150V for 60 minutes. Wet transfer was performed on nitrocellulose membranes using NuPage transfer Buffer (Life Technologies, USA) and run at 60V for 90 minutes. Blocking and probing of the membranes was performed following manufacturer's instructions (**Table S3** for Antibodies). Membranes were washed and developed using Super Signal West Pico Chemiluminescent Substrate (Thermo Scientific, USA) and Amersham Hyperfilm ECL (Amersham, USA) as per manufacturer's instruction. Densitometric analysis was performed using Image Studio Lite Ver. 4.0 Software (LICOR Biosciences, USA) and values were normalized with loading control (β-Actin or PCNA).

Immunocytochemistry

Cell were grown on Ibi-Treat slides (Ibidi, Germany) and fixed in 4% paraformaldehyde for 10 minutes at RT. A 10 minute incubation at -20˚C in methanol allowed cell permeabilization. Following one hour of blocking in TBS/0.1% Triton-X100/5% goat serum, primary and secondary antibody staining were carried out in the blocking solution, overnight at 4˚C and for 1 hour at RT. For the list of Antibodies used, see Supplemental information (**Table S3**).

Reference:

Pfaffl, M.W. (2001). A new mathematical model for relative quantification in real-time RT-PCR. Nucleic Acids Res *29*, e45.

SUPPLEMENTAL TABLES

Table S1: Expression profile of Wnt signals, receptors and co-receptors in other model organisms

Table S2: Primers for qPCR

Table S3: Antibodies

SUPPLEMENTAL MOVIES

Movie S1: The AB+WNTi Protocol leads to development of functionally active cardiomyocytes. Movie shows beating cardiomyocytes on Day6 of the AB+WNTi Protocol.

Movie S2: The C+WNTi Protocol leads to development of functionally active cardiomyocytes. Movie shows beating cardiomyocytes on Day9 of the C+WNTi Protocol.

Table S1

GENE	RELEVANT EXPRESSION IN THE MOUSE	REFERENCES
Wnt2a	▪ Anterior lateral plate mesoderm<br>▪ Cardiac crescent (overlapping with expression of *Nkx2.5* and *Tbx5* presumably in First Heart Field)<br>▪ Foregut endoderm next to cardiac mesoderm, later restricted to pericardium and the posterior pole	(Cohen et al., 2008; Goss et al., 2009; Kemp et al., 2005; Monkley et al., 1996; Tian et al., 2010)
Wnt2b **(aka *Wnt13*)**	▪ Anterior lateral plate mesoderm<br>▪ Cardiac crescent<br>▪ Foregut endoderm next to cardiac mesoderm<br>▪ Venous pole (overlapping with expression of *Tbx18*) atrioventricular region of linear heart tube	(Goss et al., 2009; Kemp et al., 2005; Yamaguchi, 2008; Zakin et al., 1998)
Wnt3	▪ Earliest mesoderm before primitive streak formation	(Liu et al., 1999)
Wnt3a	▪ Primitive streak (overlapping with expression of *Bry*(*T*))<br>▪ Posterior embryonic mesoderm	(Takada et al., 1994; Yamaguchi, 2008)
Wnt5a	▪ Node<br>▪ Primitive streak<br>▪ Primordium of the outflow tract<br>▪ Common ventricle	(Cohen et al., 2012; Schleiffarth et al., 2007; Yamaguchi, 2008)
Wnt5b	▪ Expressed in the embryo and in the adult heart	(Gavin et al., 1990; Yamaguchi, 2008)
Wnt6	▪ Cardiac precursor cells	(Schmeckpeper et al., 2015)
Wnt8a **(aka *Wnt8*)**	▪ Primitive streak<br>▪ Tubular heart	(Jaspard et al., 2000)
Wnt9b **(aka *Wnt15*)**	▪ Epicardium<br>▪ Coronary vasculature	(Merki et al., 2005)
Wnt11	▪ Node<br>▪ Cardiac crescent (FHF/SHF?)<br>▪ Linear heart tube<br>▪ OFT myocardium<br>▪ Ventricle	(Nagy et al., 2010; Terami et al., 2004; Yamaguchi, 2008)
Fzd2	▪ Cardiac Neural Crest Cells	(van Gijn et al., 2001)
Fzd4	▪ No information on expression, but associated with human heart and great vessel patterning defects	(DeRossi et al., 2000)
Fzd5	▪ Distal, then anterior visceral endoderm<br>▪ Anterior mesendoderm	(Kemp et al., 2007)
Fzd6	▪ Notochord<br>▪ Foregut endoderm	(Borello et al., 1999)
Fzd7	▪ Wide expression domain including common cardiac progenitor	(Kemp et al., 2007; Sagara et al., 1998)
Fzd9	▪ Embryonic and adult heart and cava veins	(Wang et al., 1999)
Ror1	▪ Myocardium<br>▪ Interventricular septum<br>▪ Aortic valve and atrium, but not aortic arch or in the epicardium	(Al-Shawi et al., 2001; Matsuda et al., 2001)
Ror2	▪ Myocardium<br>▪ Interventricular septum<br>▪ Aortic valve and atrium, but not aortic arch or in the epicardium	(Al-Shawi et al., 2001; Matsuda et al., 2001)
Lrp5	▪ Embryonic heart<br>▪ Atrium and ventricle	(Visel et al., 2004)

Table S1

References:

Al-Shawi, R., Ashton, S.V., Underwood, C., and Simons, J.P. (2001). Expression of the Ror1 and Ror2 receptor tyrosine kinase genes during mouse development. Development genes and evolution *211*, 161-171.

Borello, U., Buffa, V., Sonnino, C., Melchionna, R., Vivarelli, E., and Cossu, G. (1999). Differential expression of the Wnt putative receptors Frizzled during mouse somitogenesis. Mechanisms of development *89*, 173-177.

Cohen, E.D., Miller, M.F., Wang, Z., Moon, R.T., and Morrisey, E.E. (2012). Wnt5a and Wnt11 are essential for second heart field progenitor development. Development *139*, 1931-1940.

Cohen, E.D., Tian, Y., and Morrisey, E.E. (2008). Wnt signaling: an essential regulator of cardiovascular differentiation, morphogenesis and progenitor self-renewal. Development *135*, 789-798.

DeRossi, C., Laiosa, M.D., Silverstone, A.E., and Holdener, B.C. (2000). Mouse fzd4 maps within a region of chromosome 7 important for thymus and cardiac development. Genesis *27*, 64-75.

Gavin, B.J., McMahon, J.A., and McMahon, A.P. (1990). Expression of multiple novel Wnt-1/int-1-related genes during fetal and adult mouse development. Genes & development *4*, 2319-2332.

Goss, A.M., Tian, Y., Tsukiyama, T., Cohen, E.D., Zhou, D., Lu, M.M., Yamaguchi, T.P., and Morrisey, E.E. (2009). Wnt2/2b and beta-catenin signaling are necessary and sufficient to specify lung progenitors in the foregut. Developmental cell *17*, 290-298.

Jaspard, B., Couffinhal, T., Dufourcq, P., Moreau, C., and Duplaa, C. (2000). Expression pattern of mouse sFRP-1 and mWnt-8 gene during heart morphogenesis. Mechanisms of development *90*, 263-267.

Kemp, C., Willems, E., Abdo, S., Lambiv, L., and Leyns, L. (2005). Expression of all Wnt genes and their secreted antagonists during mouse blastocyst and postimplantation development. Developmental dynamics : an official publication of the American Association of Anatomists *233*, 1064-1075.

Kemp, C.R., Willems, E., Wawrzak, D., Hendrickx, M., Agbor Agbor, T., and Leyns, L. (2007). Expression of Frizzled5, Frizzled7, and Frizzled10 during early mouse development and interactions with canonical Wnt signaling. Developmental dynamics : an official publication of the American Association of Anatomists *236*, 2011-2019.

Liu, P., Wakamiya, M., Shea, M.J., Albrecht, U., Behringer, R.R., and Bradley, A. (1999). Requirement for Wnt3 in vertebrate axis formation. Nature genetics *22*, 361-365.

Matsuda, T., Nomi, M., Ikeya, M., Kani, S., Oishi, I., Terashima, T., Takada, S., and Minami, Y. (2001). Expression of the receptor tyrosine kinase genes, Ror1 and Ror2, during mouse development. Mechanisms of development *105*, 153-156.

Merki, E., Zamora, M., Raya, A., Kawakami, Y., Wang, J., Zhang, X., Burch, J., Kubalak, S.W., Kaliman, P., Izpisua Belmonte, J.C., *et al.* (2005). Epicardial retinoid X receptor alpha is required for myocardial growth and coronary artery formation. Proceedings of the National Academy of Sciences of the United States of America *102*, 18455-18460.

Monkley, S.J., Delaney, S.J., Pennisi, D.J., Christiansen, J.H., and Wainwright, B.J. (1996). Targeted disruption of the Wnt2 gene results in placentation defects. Development *122*, 3343-3353.

Nagy, II, Railo, A., Rapila, R., Hast, T., Sormunen, R., Tavi, P., Rasanen, J., and Vainio, S.J. (2010). Wnt-11 signalling controls ventricular myocardium development by patterning N-cadherin and beta-catenin expression. Cardiovascular research *85*, 100-109.

Table S1

Sagara, N., Toda, G., Hirai, M., Terada, M., and Katoh, M. (1998). Molecular cloning, differential expression, and chromosomal localization of human frizzled-1, frizzled-2, and frizzled-7. Biochemical and biophysical research communications *252*, 117-122.

Schleiffarth, J.R., Person, A.D., Martinsen, B.J., Sukovich, D.J., Neumann, A., Baker, C.V., Lohr, J.L., Cornfield, D.N., Ekker, S.C., and Petryk, A. (2007). Wnt5a is required for cardiac outflow tract septation in mice. Pediatric research *61*, 386-391.

Schmeckpeper, J., Verma, A., Yin, L., Beigi, F., Zhang, L., Payne, A., Zhang, Z., Pratt, R.E., Dzau, V.J., and Mirotsou, M. (2015). Inhibition of Wnt6 by Sfrp2 regulates adult cardiac progenitor cell differentiation by differential modulation of Wnt pathways. Journal of molecular and cellular cardiology *85*, 215-225.

Takada, S., Stark, K.L., Shea, M.J., Vassileva, G., McMahon, J.A., and McMahon, A.P. (1994). Wnt-3a regulates somite and tailbud formation in the mouse embryo. Genes & development *8*, 174-189.

Terami, H., Hidaka, K., Katsumata, T., Iio, A., and Morisaki, T. (2004). Wnt11 facilitates embryonic stem cell differentiation to Nkx2.5-positive cardiomyocytes. Biochemical and biophysical research communications *325*, 968-975.

Tian, Y., Cohen, E.D., and Morrisey, E.E. (2010). The importance of Wnt signaling in cardiovascular development. Pediatric cardiology *31*, 342-348.

van Gijn, M.E., Blankesteijn, W.M., Smits, J.F., Hierck, B., and Gittenberger-de Groot, A.C. (2001). Frizzled 2 is transiently expressed in neural crest-containing areas during development of the heart and great arteries in the mouse. Anatomy and embryology *203*, 185-192.

Visel, A., Thaller, C., and Eichele, G. (2004). GenePaint.org: an atlas of gene expression patterns in the mouse embryo. Nucleic acids research *32*, D552-556.

Wang, Y.K., Sporle, R., Paperna, T., Schughart, K., and Francke, U. (1999). Characterization and expression pattern of the frizzled gene Fzd9, the mouse homolog of FZD9 which is deleted in Williams-Beuren syndrome. Genomics *57*, 235-248.

Yamaguchi, T.P. (2008). Genetics of Wnt signaling during early mammalian development. Methods Mol Biol *468*, 287-305.

Zakin, L.D., Mazan, S., Maury, M., Martin, N., Guenet, J.L., and Brulet, P. (1998). Structure and expression of Wnt13, a novel mouse Wnt2 related gene. Mechanisms of development *73*, 107-116.

TABLE S2: qPCR PRIMERS

Primers for RT-qPCR(Genes)	Sequences (5’-3’)
GAPDH	F: AATTCCATGGCACCGTCAAG<br>R: ATCGCCCCACTTGATTTTGG
SOX2	F: ATGACCAGCTCGCAGACCTA<br>R: GACTTGACCACCGAACCCAT
Bry (T)	F: GCTGTGACAGGTACCCAACC<br>R: CATGCAGGTGAGTTGTCAGAA
MESP1	F: TAGGGCTAGACACTTTGAG<br>R: TTTTTGCCAACTGACACC
MIXL1	F: GGTACCCCGACATCCACTT<br>R: TGGAAGGATTTCCCACTCTG
MSX1	F: GAGTTCTCCAGCTCGCTCA<br>R: CCAGCTCTGCCTCTTGTAGTC
NKX2.5	F: CAAGTGTGCGTCTGCCTTTC<br>R: CGCGCACAGCTCTTTCTTTT
TBX5	F: GGAGCTGCACAGAATGTCAA<br>R: TGCTGAAAGGACTGTGGTTG
cTnT	F: TCCCCGATGGAGAGAGAGTG<br>R: ACGAGCTCCTCCTCCTCTTT
MYH6	F: CTCAAGCTCATGGCCACTCT<br>R: GCCTCCTTTGCTTTTACCACT
ACTN2	F: CTGCTGCTTTGGTGTCAGAG<br>R: TTCCTATGGGGTCATCCTTG
AXIN2	F: GTGAGGTCCACGGAAACTGT<br>R: TGGCTGGTGCAAAGACATAG
DKK1	F: CCTTGGATGGGTATTCCAGA<br>R: CCTGAGGCACAGTCTGATGA
ALCAM	F: TAACTTGCACAGCAGAAAAC<br>R: ATGCAGTCTTTGACTTCTTC
HCN4	F: GATCCTCAGCCTCTTACGCC<br>R: AGGAGCATCATGCCGATGAG
WNT1	F: CGGCGTTTATCTTCGCTATC<br>R: TTCGATGGAACCTTCTGAGC
WNT2	F: AAAGAAGATGGGAAGCGCCA<br>R: TTCATCAGGGCTCTGGCATC
WNT2B	F: CCGAGAGTGTCAGCACCAAT<br>R: TGGACTACCCCTGCTGATGA
WNT3	F: CGCACGACTATCCTGGAC<br>R: GAGGCGCTGTCATACTTGTC
WNT3A	F: TGTTGGGCCACAGTATTCCT<br>R: GGGCATGATCTCCACGTAGT
WNT4	F: CTAGCCCCGACTTCTGTGAG<br>R: TTGGACGTCTTGTTGCATGT
WNT5A	F: GCCCAGGTTGTAATTGAAGC<br>R: TGGCACAGTTTCTTCTGTCC
WNT5B	F: CGGGAGCGAGAGAAGAACT<br>R: CGTCTGCCATCTTATACACAGC

WNT6	F: CAGCCCCTTGGTTATGGAC<br>R: AACTGGAACTGGCACTCTCG
WNT7A	F: AGAAGCAAGGCCAGTACCAC<br>R: GCCTCGTTGTTGTGCAAGTT
WNT7B	F: GCAGGAAGGTTCTAGAGG<br>R: GTTGTACTTCTCCTTCAGC
WNT8A	F: AGGCTGAGAAGTGCTACCAGA<br>R: CCATTGTTTGACCCATCACA
WNT8B	F: GAAGTACCACGCAGCACTCA<br>R: GAGATGGAGCGAAAGGTGTC
WNT9A	F: GGTGTGAAGGTGATCAAG<br>R: TGCCGTCTCATACTTGTG
WNT9B	F: AGAGGAAACAAGGACCTGCG<br>R: GCACTTACACGTGGTCCTGA
WNT10A	F: CATCTTCAGCAGAGGTTTC<br>R: CAGTGCATCCAGTTGTAAG
WNT10B	F: GATACCCACAACCGCAATTC<br>R: GGGTCTCGCTCACAGAAGTC
WNT11	F: GGCCAAGTTTTCCGATGCTC<br>R:CACCCCATGGCACTTACACT
WNT16	F: GAAACTGGATGTGGTTGG<br>R:TCATGCAGTTCCATCTCTC
FZD1	F: CATCGTCATCGCCTGCTACT<br>R: TAGCGTAGCTCTTGCAGCTC
FZD2	F: CTTCTCACAGGAGGAGACGC<br>R; AAATGATAGGCCGCTCTGGG
FZD3	F: ACAGCAAAGTGAGCAGCTACC<br>R: CTGTAACTGCAGGGCGTGTA
FZD4	F: TCAAGAGACGCTGTGAACCC<br>R: GGTCGTTCTGTGGTGGGAAT
FZD5	F: GCACAACCACATCCACTACG<br>R: GCCATGCCGAAGAAGTAGAC
FZD6	F: AGGCTTGCACCGTTTTGTTC<br>R: TGCTCGATGGCTTCACAACT
FZD7	F: CGCCTCTGTTCGTCTACCTC<br>R: TCATGATGGTGCGGATACGG
FZD8	F: CGCCTCTGTTCGTCTACCTC<br>R: TCATGATGGTGCGGATACGG
FZD9	F: TGCTCACCTTCTTGCTGGAG<br>R: GCCAGCGAGTAGACGTTGTA
FZD10	F: AAGAAGAGCCGGAGAAAACC<br>R: GACTGGGCAGGGATCTCATA
LRP5	F: CACCACCTTCTTGCTGTTCA<br>R: GCTTTGACGTTCCTCAGTCC
LRP6	F: GACTGGGTTGCACGAAATCT<br>R: CGGGGTTCCTCTAAGTCCTC
RYK	F: CCGCATACCTGAAAGATGGT<br>R: GATCTAAGGCCCAGCAACAG
ROR2	F: CCCCTCATTAACCAGCACAA<br>R: TTCCCAAACCGGTCCTCT

TABLE S3: ANTIBODIES

Primary Antibodies				
Antibody reactivity	Dilution	Application	Manufacturer	Catalogue #
OCT4	1/500	ICC	Abcam	Ab137427
Bry (T)	1/50	ICC	Abcam	Ab20680
NKX2.5	1/800	ICC	Cell Signalling Technology	8792S
MYH6	1/100	ICC	Proteintech	22281-1-AP
Active Beta Catenin	1/500<br>1/1000	ICC<br>WB	Millipore	05-665
Phospho-SAPK/JNK	1/400	ICC	Cell Signalling Technology	9255S
DAPI	1/1000	ICC	Thermo Scientific	62248
Phospho-SAPK/JNK	1/500	WB	Cell Signalling Technology	9255S
Beta Catenin	1/1000	WB	Santa Cruz	SC-7199
SAPK/JNK	1/1000	WB	Cell Signalling Technology	9251S
Beta Actin	1/1000	WB	Cell Signalling Technology	4970S
**Secondary Antibodies**				
Antibody reactivity	Dilution	Application	Manufacturer	Catalogue #
Goat anti-Rabbit IgG (H+L) Alexa Fluor® 488 conjugate	1/400	ICC	Thermo Scientific	A-11008
Goat anti-Mouse IgG (H+L) Alexa Fluor® 488 conjugate	1/400	ICC	Thermo Scientific	A-11001
Anti-rabbit IgG, HRP-linked	1/10000	WB	Cell Signalling Technology	7074
Anti-mouse IgG, HRP-linked	1/10000	WB	Cell Signalling Technology	7076
