# Supplementary figures and images for: Distinctive Roles of Canonical and Noncanonical Wnt Signaling in Human Embryonic Cardiomyocyte Development
Source: Stem Cell Reports. 2016 Sep 15;7(4):764–76. doi: 10.1016/j.stemcr.2016.08.008 (PMC5063467; doi:10.1016/j.stemcr.2016.08.008)

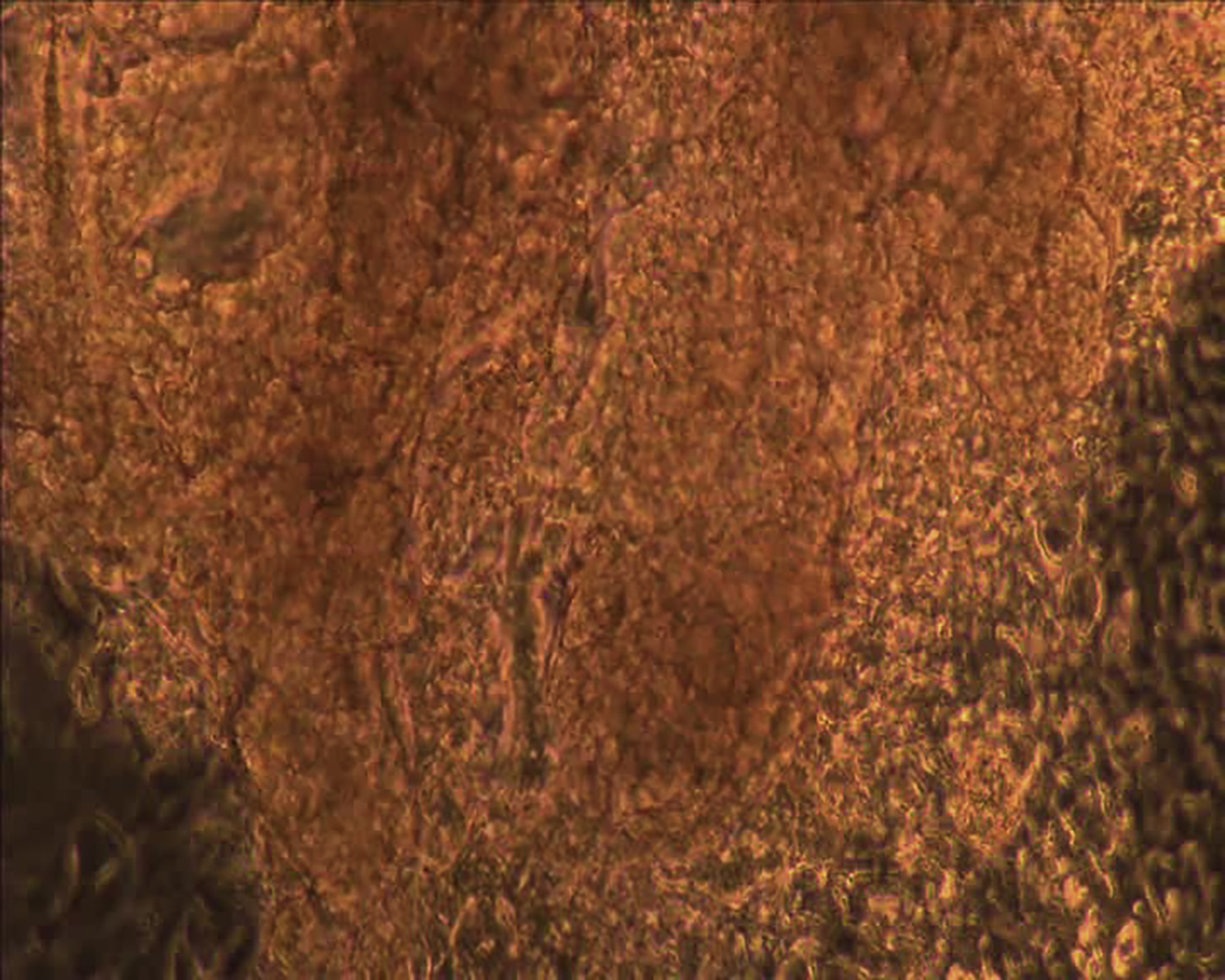

Supplement: Movie S1. The AB + WNTi Protocol Leads to Development of Functionally Active Cardiomyocytes [file mmc2.jpg]

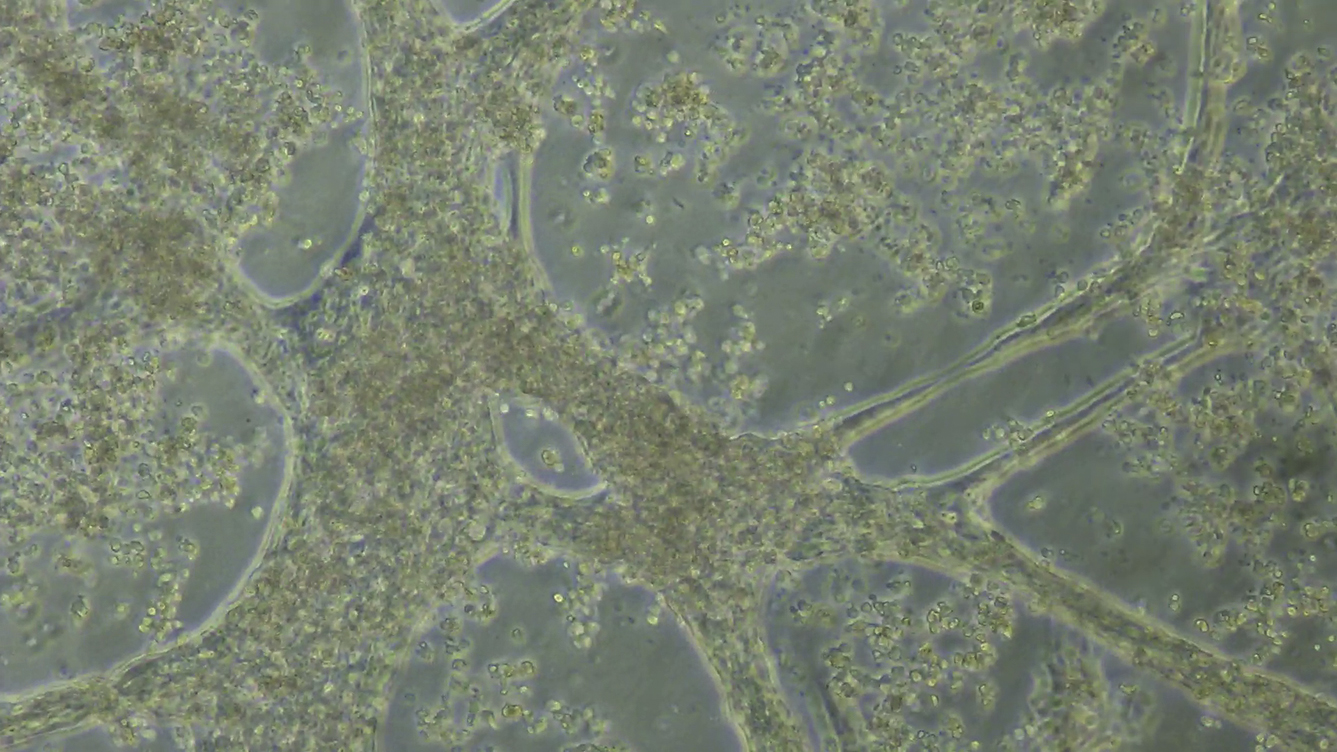

Supplement: Movie S2. The C + WNTi Protocol Leads to Development of Functionally Active Cardiomyocytes [file mmc3.jpg]
