# Supplementary material for: Distinctive Roles of Canonical and Noncanonical Wnt Signaling in Human Embryonic Cardiomyocyte Development
Source: Stem Cell Reports. 2016 Sep 15;7(4):764–76. doi: 10.1016/j.stemcr.2016.08.008 (PMC5063467; doi:10.1016/j.stemcr.2016.08.008)
Supplement: Document S2. Article plus Supplemental Information [file mmc4.pdf]

Stem Cell Reports
Article

ISSCR

OPEN ACCESS

Distinctive Roles of Canonical and Noncanonical Wnt Signaling in Human Embryonic Cardiomyocyte Development

Silvia Mazzotta,[1] Carlos Neves,[1] Rory J. Bonner,[1] Andreia S. Bernardo,[2,3] Kevin Docherty,[1] and Stefan Hoppler[1,*]
[1]Institute of Medical Sciences, University of Aberdeen, Aberdeen AB25 2ZD, UK
[2]The Francis Crick Institute, Mill Hill Laboratory, London NW7 1AA, UK
[3]Anne McLaren Laboratory for Regenerative Medicine, University of Cambridge, West Forvie Building, Robinson Way, Cambridge CB2 0SZ, UK
*Correspondence: s.p.hoppler@abdn.ac.uk
http://dx.doi.org/10.1016/j.stemcr.2016.08.008

SUMMARY

Wnt signaling is a key regulator of vertebrate heart development; however, specific roles for human cardiomyocyte development remain uncertain. Here we use human embryonic stem cells (hESCs) to analyze systematically in human cardiomyocyte development the expression of endogenous Wnt signaling components, monitor pathway activity, and dissect stage-specific requirements for canonical and noncanonical Wnt signaling mechanisms using small-molecule inhibitors. Our analysis suggests that WNT3 and WNT8A, via FZD7 and canonical signaling, regulate *BRACHYURY* expression and mesoderm induction; that WNT5A/5B, via ROR2 and noncanonical signaling, regulate *MESP1* expression and cardiovascular development; and that later in development WNT2, WNT5A/5B, and WNT11, via FZD4 and FZD6, regulate functional cardiomyocyte differentiation via noncanonical Wnt signaling. Our findings confirm in human development previously proposed roles for canonical Wnt signaling in sequential stages of vertebrate cardiomyogenesis, and identify more precise roles for noncanonical signaling and for individual Wnt signal and Wnt receptor genes in human cardiomyocyte development.

INTRODUCTION

The human heart develops as the first functional organ in the embryo. In the lateral plate mesoderm (LPM) cardiac progenitor cells are induced, which can subsequently differentiate into heart muscle cells (cardiomyocytes) (Rosenthal and Harvey, 2010; Sylva et al., 2014). Wnt signaling, a well-known key regulator of vertebrate cardiomyocyte differentiation (Hoppler et al., 2014), acts through several molecular mechanisms (Hoppler and Nakamura, 2014): a β-catenin-dependent, so-called canonical pathway, and β-catenin-independent, so-called noncanonical pathways, among which a JNK-dependent pathway is prominent during heart development (Gessert and Kühl, 2010; Gessert et al., 2008; Pandur et al., 2002a, 2002b).

Several studies in mouse and other experimental models have described diverse, and often opposing, effects of canonical and noncanonical Wnt signaling on subsequent cardiomyocyte differentiation, leading to the argument that particularly the JNK-mediated noncanonical pathway may function in this context to antagonize canonical Wnt signaling (Abdul-Ghani et al., 2011; Cohen et al., 2012; reviewed by Hoppler et al., 2014). In addition, canonical Wnt signaling has been shown to play multiple and conflicting roles at different stages of heart development (Gessert and Kühl, 2010; Naito et al., 2006). However, specific roles for Wnt signaling in human cardiomyocyte development remain unclear, particularly regarding which endogenous Wnt ligands and Wnt receptors are involved.

In this study we therefore used human embryonic stem cells (hESCs) to dissect stage-specific requirements of Wnt signaling and to identify endogenous Wnt ligand and receptor genes directing human embryonic cardiomyocyte development.

RESULTS

Investigating Wnt Signaling during Human Cardiomyocyte Development Using hESCs

To study sequential stages of human cardiomyocyte development in vitro, we explored established hESC differentiation protocols. With two hESC cell lines (H9 and H7) we used an activin/bone morphogenetic protein (BMP)-based protocol, hereby named the AB + WNTi protocol (Figure 1A and Experimental Procedures, modified from Mendjan et al., 2014), and a CHIR99021-based protocol, hereby named C + WNTi (Figure 1B and Experimental Procedures, modified from Lian et al., 2013). As previously shown, these protocols lead to loss of pluripotency, induction of mesoderm identity, development of cardiac precursor identity in an LPM context, and differentiation into beating primary cardiomyocytes (Figures S1C–S1F). These protocols are thereby clearly following the normal developmental progress in the embryonic heart (Rosenthal and Harvey, 2010; Sylva et al., 2014). Beating activity is observed as early as day 6 for the AB + WNTi protocol (Movie S1) and day 9 for the C + WNTi protocol (Movie S2).

This is an open access article under the CC BY license (http://creativecommons.org/licenses/by/4.0/).

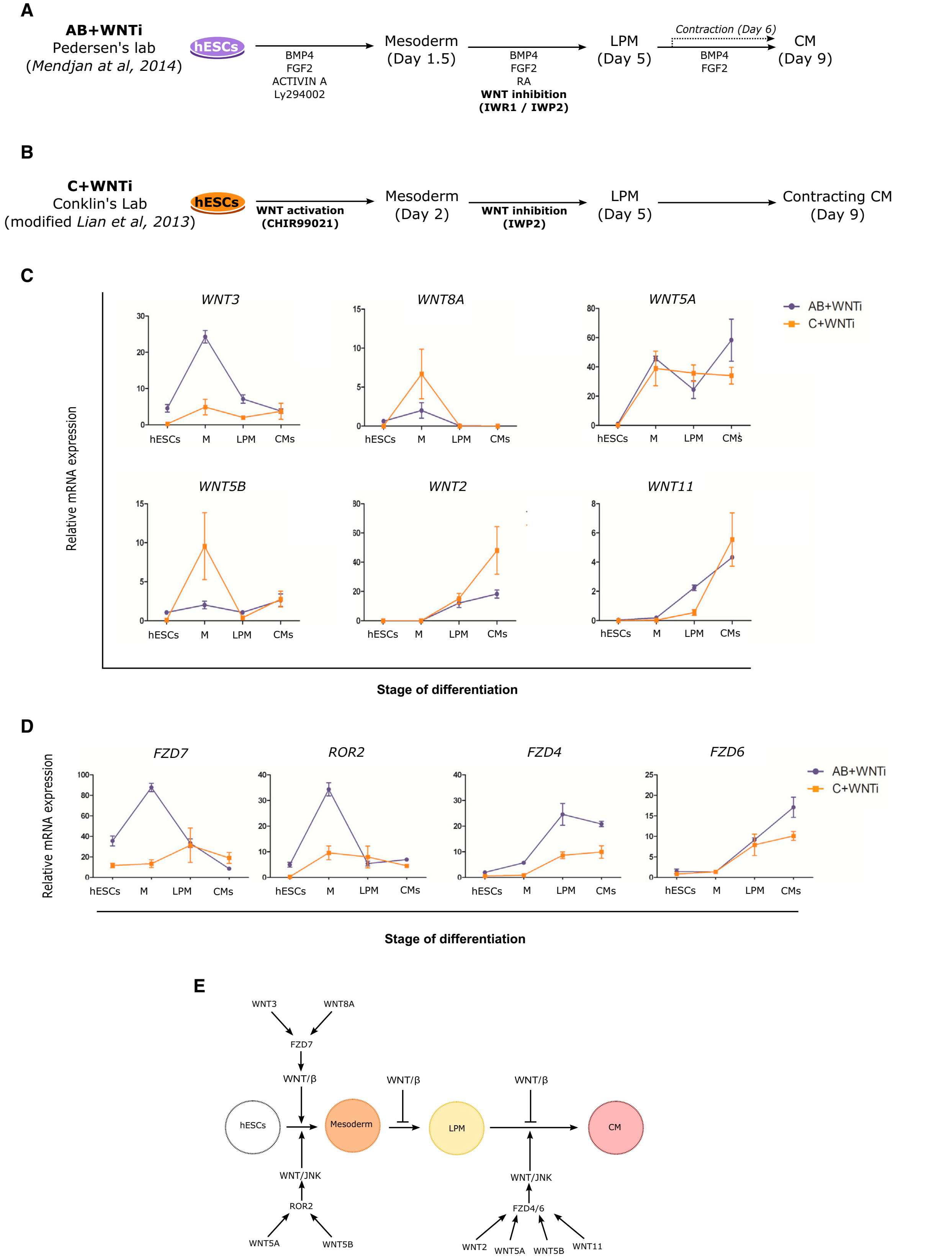

(legend on next page)

Consistent with findings from model systems (e.g., Singh et al., 2007; Ueno et al., 2007), inhibition of canonical Wnt signaling following mesoderm induction is essential for efficient subsequent human cardiomyocyte differentiation (Figures S1A and S1B) (see also Burridge et al., 2014; Cao et al., 2013; Laflamme et al., 2007; Lian et al., 2012; Mendjan et al., 2014; Paige et al., 2010; Willems et al., 2011). In this study we mainly use the small-molecule Wnt inhibitor IWP2 (an inhibitor of Wnt ligand secretion which, as such, interferes with any Wnt-mediated signaling, irrespective of pathway mechanisms) and also IWR1 (a specific inhibitor of canonical Wnt/β-catenin signaling) (Chen et al., 2009). Either inhibitor promotes subsequent cardiomyocyte differentiation, confirming that inhibition of canonical Wnt signaling is sufficient in this context for differentiation of hESCs into functional cardiomyocytes (Figure S1).

Endogenous Expression of Wnt Signaling Ligands and Receptors Suggests Stage-Specific Roles for Wnt Signaling in Cardiomyocyte Differentiation

Manipulation of Wnt signaling is a well-established experimental tool for driving cardiomyocyte differentiation in hESC protocols (e.g., Lian et al., 2012), suggesting a key role for Wnt pathways in endogenous cardiomyocyte development. So far, information about specific Wnt signals and receptors is only available from animal models (Table S1; Eisenberg and Eisenberg, 1999; Hardy et al., 2008; Kilian et al., 2003; Lavery et al., 2008; Pandur et al., 2002a; Ulrich et al., 2005). To identify WNT signals and receptors that may regulate cardiomyocyte development in humans, we systematically studied the expression of all 19 genes encoding WNT signals (Figure S2A), of all ten frizzled (FZD) receptors, and of WNT co-receptors at key stages of human cardiomyocyte development (Figure S2B), using the aforementioned protocols.

We discovered that *WNT3* and *WNT5A* are particularly highly expressed during mesoderm induction, together with *WNT8A* and *WNT5B*. Much later in development, during cardiomyocyte differentiation, high expression of *WNT2*, *WNT5A*, *WNT5B*, and *WNT11* was observed (Figures 1C and S2A). We also uncovered strong expression of both *FZD7* and *ROR2* during mesoderm induction, and *FZD4* and *FZD6* expression during cardiomyocyte differentiation (Figures 1D and S2B). *WNT3* and *WNT8A* are known to activate the canonical Wnt pathway (Yamaguchi, 2008) while *WNT5A*, *WNT5B*, and *WNT11* are often associated with noncanonical Wnt signaling mechanisms (Cohen et al., 2012; He et al., 1997; Pandur et al., 2002a; Yamaguchi, 2008).

These results allowed us to formulate an initial working hypothesis (Figure 1E) proposing that canonical Wnt signaling is activated during mesoderm induction by WNT3 and WNT8A, via FZD7; and that JNK-mediated noncanonical Wnt signaling is activated by WNT5A and WNT5B, probably involving ROR2. During cardiomyocyte differentiation stages, the expression of *WNT5A*, *WNT5B*, and *WNT11* suggests that these ligands activate noncanonical Wnt signaling via *FZD4* and *FZD6*, and possibly together with *WNT2*, presumably through a JNK-mediated mechanism as previously suggested in the mouse system (Gessert and Kühl, 2010; Pandur et al., 2002a). These proposed functions of human genes in this working hypothesis are on the whole consistent with the expression of the mouse homologs, where such information is available (Table S1). However, we proceeded to test further predictions of this working hypothesis.

Canonical and Noncanonical Wnt Pathway Activation during Different Stages of Human Cardiomyocyte Differentiation

To test our working hypothesis (Figure 1E) and further explore this model, we studied Wnt signaling pathway

Figure 1. *WNT* Signal and Receptor Gene Expression in Human Cardiomyocyte Development Using hESC Differentiation Protocols
(A) Schematic description of the cardiomyocyte differentiation protocol AB + WNTi. This established protocol guides pluripotent hESC first through mesoderm (M) induction with reagents including activin A and BMP4, AB, and then drives lateral plate mesoderm (LPM) and ultimately cardiomyocyte (CM) development with reagents including Wnt inhibitors, WNTi (IWR1 or IWP2).
(B) Schematic description of the cardiomyocyte differentiation protocol C + WNTi. This established protocol drives mesoderm induction by adding the Wnt signaling agonist CHIR99021, C, and then promotes cardiomyocyte development with Wnt signaling inhibitors, WNTi (i.e., IWP2).
(C) *WNT* gene expression in human cardiomyocyte differentiation protocols by qPCR analysis. Note the prominent expression of *WNT3*, *8A*, *5A*, and *5B* early during mesoderm induction and expression of *WNT5A*, *5B*, *2*, and *11* later during cardiomyocyte differentiation.
(D) *FZD* receptor gene and WNT co-receptor gene expression in human cardiomyocyte differentiation protocols analyzed by qPCR. Note the prominent expression of *FZD7* and *ROR2* early during mesoderm induction and *FZD4* and *FZD6* later during cardiomyocyte differentiation.
(E) Schematic representation of the working hypothesis about Wnt signaling mechanisms regulating human cardiomyocyte development early during mesoderm induction and later during cardiomyocyte differentiation.
qPCR data are presented as means ± SEM for n = 3 independent experiments. *GAPDH* was used as a housekeeping gene. See Experimental Procedures for further details of cardiomyocyte differentiation protocols. See Figure S2 for comprehensive expression analysis of all *WNT* genes, all *FZD* genes, and several WNT co-receptor genes, and Table S1 for information on spatial expression of mouse homologs.

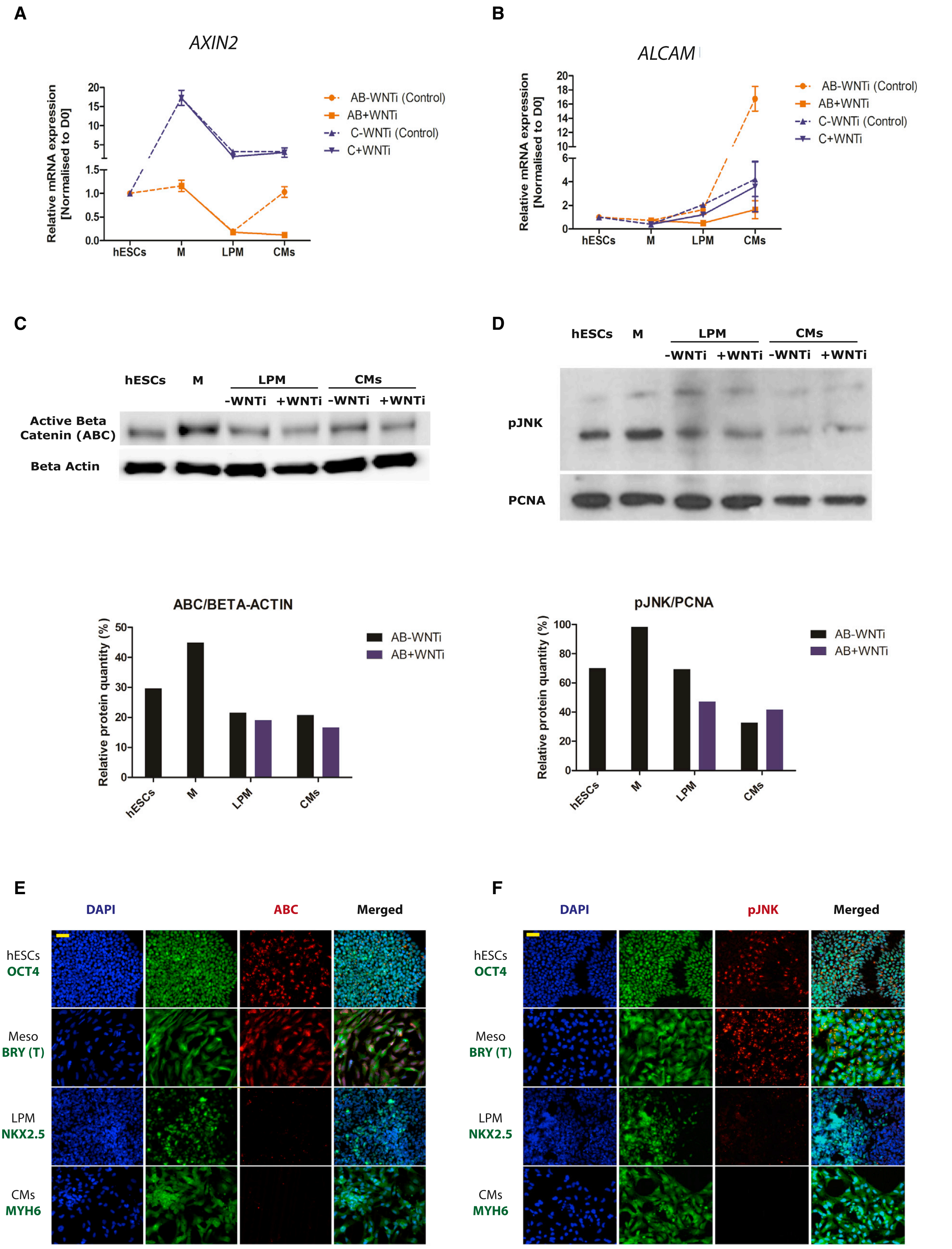

(legend on next page)

activity during sequential stages of the cardiomyocyte differentiation protocols.

Canonical Wnt signaling activity was monitored by following the expression levels of the known Wnt/β-catenin target gene *AXIN2* (Yan et al., 2001) and by analyzing protein abundance and localization of active β-catenin (ABC) (Staal et al., 2002). Our molecular understanding of noncanonical WNT signaling is still rudimentary, and methods for monitoring noncanonical Wnt signaling activity are therefore less well established; specifically, there is no reliable pathway indicator gene for noncanonical WNT signaling. Since a JNK-dependent Wnt pathway is prominent during heart development (Gessert and Kühl, 2010; Hoppler et al., 2014), we monitored expression of the recently identified target gene of noncanonical Wnt/JNK signaling, *ALCAM* (Choudhry and Trede, 2013; Cizelsky et al., 2014), and protein abundance and cellular localization of phosphorylated JNK (Pandur et al., 2002a).

As predicted by the working hypothesis (Figure 1E), we find that *AXIN2* expression is high during mesoderm induction (Figure 2A), and consistently the ABC protein is enriched (Figure 2C) and has a nuclear localization in induced mesoderm (Figure 2E), demonstrating active canonical Wnt signaling during these early stages. Contrarily, *AXIN2* expression levels clearly reveal that canonical Wnt signaling is reduced after mesoderm induction in these human cardiomyocyte differentiation protocols, and remains reduced during cardiomyocyte differentiation (Figure 2A). These findings are further supported by the low levels of ABC protein at these later stages (Figures 2C and 2E).

Consistent with the working hypothesis (Figure 1E), we find activation of JNK-mediated signaling during mesoderm induction when analyzing phosphorylated JNK protein levels and cellular localization (Figures 2D and 2F), and *ALCAM* expression gradually increasing at later stages (Figure 2B). However, *ALCAM* is not expressed during mesoderm induction and might therefore not be a good indicator of noncanonical Wnt/JNK signaling at this stage. At later stages analysis of phosphorylated JNK did not consistently indicate an increase, which would be contrary to the prediction of the working hypothesis and the ALCAM expression data. However, as expected, addition of IWP2 reduces *ALCAM* expression and JNK phosphorylation.

Altogether, these data support our working hypothesis (Figure 1E), as far as showing that canonical Wnt signaling is confined to mesoderm induction, and suggest potential noncanonical Wnt signaling activity during mesoderm induction and cardiomyocyte differentiation. We therefore proceeded to further test our working hypothesis by experimentally interfering with the Wnt pathway at critical stages of cardiomyocyte development.

Both β-Catenin- and JNK-Mediated Signaling Are Required Early during Mesoderm Development

To investigate any requirement for canonical and noncanonical Wnt signaling during mesoderm induction, we manipulated the AB + WNTi protocol by adding at this stage the small molecules IWP2, IWR1, or JNKi (Figure 3A). IWP2 is used as above to interfere with Wnt signaling in general, and IWR1 as a specific inhibitor of canonical Wnt/β-catenin signaling (Chen et al., 2009). JNKi (SP600125) is used to inhibit JNK-dependent noncanonical Wnt signaling (Pandur et al., 2002a).

Mesoderm induction is characterized by a dramatic change of cell morphology, from round and small hESCs tightly organized in colonies, to fibroblastoid mesodermal cells that are well spread out from the original colony. Experimental inhibition of either Wnt pathway clearly interfered with these morphological rearrangements (Figure 3B) and, therefore, mesoderm induction.

Figure 2. Activity of Wnt Signaling Pathways in Human Cardiomyocyte Differentiation Protocols

(A) Gene expression (analyzed by qPCR) of the canonical Wnt target gene *AXIN2*. Note *AXIN2* expression confined to mesoderm induction and reduced expression during cardiomyocyte differentiation.

(B) Gene expression (analyzed by qPCR) of the proposed noncanonical Wnt target gene *ALCAM*. Note *ALCAM* expression only increasing at later stages of the protocol, but decreasing following addition of Wnt inhibitor.

(C) Top panel: western blot analysis of active β-catenin (ABC) and β-actin in the AB −/+ WNTi (i.e., IWP2) protocols. Bottom panel: quantified western analysis showing protein abundance of ABC relative to the housekeeping protein β-actin. Note increased ABC abundance confined to mesoderm induction.

(D) Top panel: western blot analysis of phosphorylated JNK (pJNK) and proliferating cell nuclear antigen (PCNA) in the AB −/+ WNTi (i.e., IWP2) protocols. Bottom panel: quantified western analysis showing protein abundance of pJNK relative to the housekeeping protein PCNA. Note increased pJNK abundance mostly confined to mesoderm induction.

(E) Tissue-wide analysis of Wnt/β-catenin pathway activity. Immunocytochemistry analysis of activated β-catenin (ABC) at different stages of cardiomyocyte differentiation protocols, marked by the localization of OCT4 (pluripotent hESCs), BRY (T) (mesoderm), NKX2.5 (LPM), and MYH6 (cardiomyocytes). Note uniform nuclear localization of ABC confined to mesoderm induction stages. Scale bar, 50 μm.

(F) Tissue-wide analysis of Wnt/JNK pathway activity. Immunocytochemistry analysis of phosphorylated JNK (pJNK) at different stages of cardiomyocyte differentiation protocols, marked by the localization of OCT4 (pluripotent hESCs), BRY (T) (mesoderm), NKX2.5 (LPM), and MYH6 (cardiomyocytes). Note uniform cytoplasmic pJNK localization early during mesoderm induction. Scale bar, 50 μm.

qPCR data are presented as means ± SEM normalized to day 0 for n = 3 independent experiments. *GAPDH* was used as a housekeeping gene.

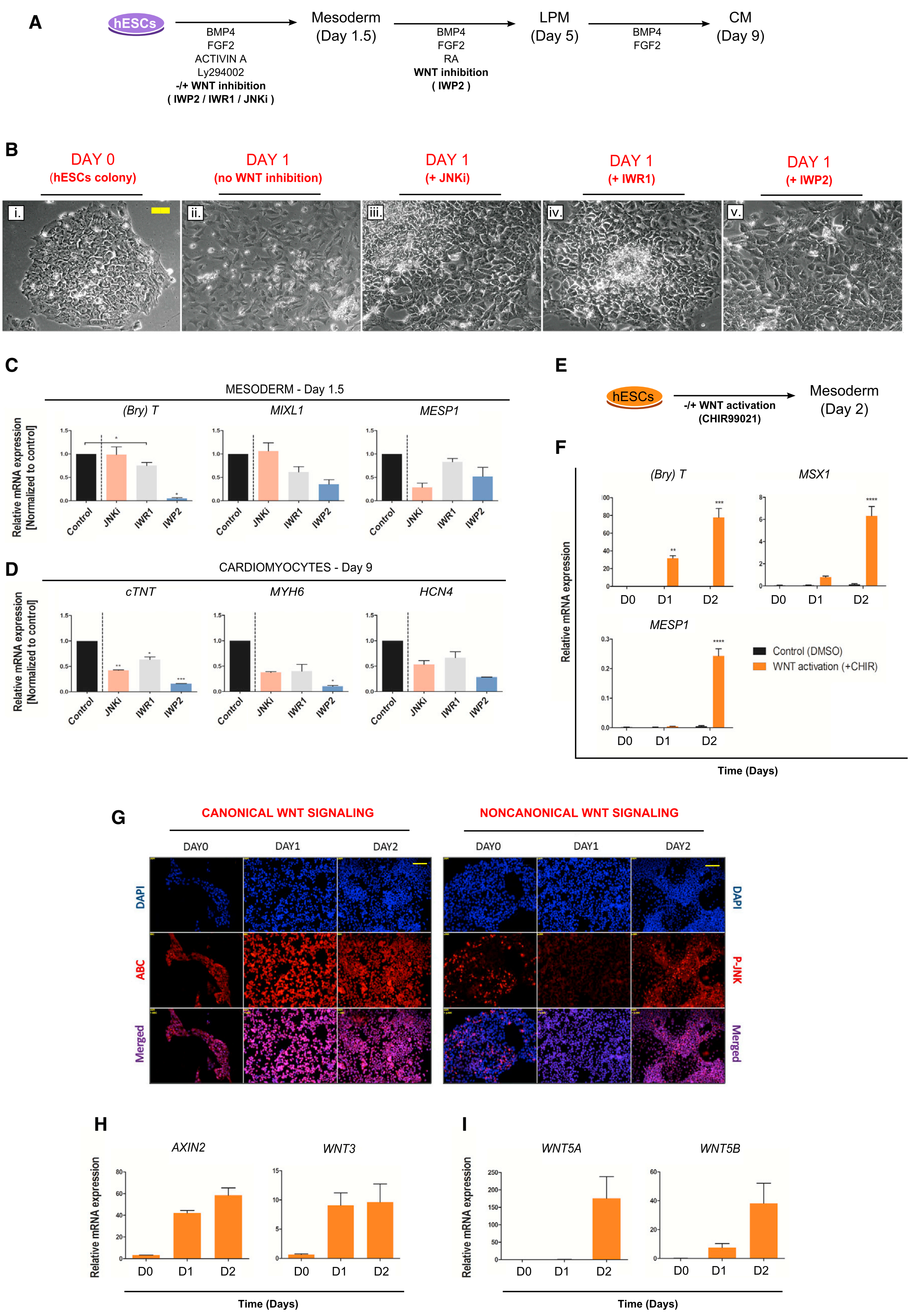

(legend on next page)

Expression of the mesodermal markers *BRACHYURY* (*BRY* [*T*]) and *MIXL1* is found to be reduced following inhibition of canonical Wnt signaling in the AB + WNTi protocol, but only the expression of the earliest cardiogenic marker *MESP1* (David et al., 2008) is decreased following inhibition of noncanonical WNT/JNK signaling (Figure 3C). These effects on mesoderm formation lead to expected consequences later, particularly reduced levels of expression of the cardiac markers *cTNT*, *MYH6*, and *HCN4* (Figure 3D). These data suggest that both branches of Wnt signaling are required during initial mesodermal commitment; however, canonical and noncanonical Wnt signaling play different roles at this early stage.

To study any sufficiency for canonical WNT signaling in this context, we used the C + WNTi protocol. Experimental activation of canonical Wnt signaling (Figure 3E) with CHIR99021 leads to expression of *BRY* (*T*) and *MSX1* (starting on experimental day 1) and more delayed *MESP1* (experimental day 2) (Figure 3F). Conversely, absence of exogenous Wnt activation results in lack of mesodermal differentiation. More detailed analysis of Wnt signaling activity at these stages shows that the Wnt/β-catenin pathway is active earlier than the JNK-dependent pathway (experimental day 1 and day 2, respectively) (Figure 3G), which is also supported by *AXIN2* and *WNT3* expression (associated with canonical Wnt signaling, Figure 3H) being earlier than *WNT5A* and *WNT5B* (associated with noncanonical Wnt signaling, Figure 3I). Together, these results show sequential activation and confirm different roles for canonical and noncanonical WNT signaling during human mesoderm induction.

Canonical and Noncanonical Wnt Signaling Have Different Roles in Late Stages of Cardiomyocyte Differentiation

Hardly anything is currently known about canonical and noncanonical Wnt signaling at later stages of human cardiomyocyte differentiation. We therefore studied molecular differentiation of cardiomyocytes when inhibition of Wnt signaling by IWP2 was experimentally prolonged (Figure 4A), and found decreased expression of cardiomyocyte differentiation markers (Figure 4B), suggesting that Wnt signaling function is required at these stages for cardiomyocyte differentiation.

We now wondered whether we could rescue cardiomyocyte differentiation by experimentally reinstating β-catenin or JNK pathway activity (Figure 4C). Activation of β-catenin signaling boosted *AXIN2* expression (Figure 4D), as expected, but caused further decreased cardiomyocyte differentiation marker expression (Figure 4E), confirming that canonical Wnt signaling at these stages is incompatible with cardiomyocyte differentiation. Activation of JNK signaling boosted *ALCAM* expression (Figure 4F), as expected, but unexpectedly did not rescue the loss of differentiation induced by IWP2 (Figure 4G).

Figure 3. Wnt Signaling Function during Mesoderm Development

(A) Schematic description of the cardiomyocyte differentiation protocol to study the requirement for Wnt signaling pathway function during mesoderm development. The protocol AB + WNTi (as in Figure 1A) is modified to inhibit Wnt signaling mechanisms (by addition of IWP2, IWR1, or JNKi [SP600125]) during mesoderm induction.

(B) Morphology of cultured cells (bright-field micrographs; scale bar, 400 μm) at the start of the differentiation protocol (day 0, panel Bi) and after 1 day (day 1, panels Bii–Bv) under different experimental conditions as shown in (A). (Bii) represents the control (no WNT inhibition); (Biii) represents cells treated with JNKi; (Biv) represents cells treated with IWR1; (Bv) represents cells treated with IWP2.

(C) Gene expression (qPCR analysis, relative to control) of markers of mesoderm induction at day 1.5 in different conditions as shown in (A). Note that JNK signaling appears to be specifically required for *MESP1* expression.

(D) Gene expression (qPCR analysis, relative to control) of cardiomyocyte markers at day 9 in the different conditions shown in (A).

(E) Schematic description of the protocol to study sufficiency in this context of Wnt signaling pathway function for mesoderm development (as in Figure 1B).

(F) Gene expression of mesoderm markers (qPCR analysis) on day 0, day 1, and day 2 in the different conditions shown in (E). Note that *MESP1* expression is induced later than *BRY* (*T*) and *MSX1*.

(G) Activity of Wnt signaling mechanisms during mesoderm development, in the presence of canonical Wnt activation (CHIR99021) as shown in (E). Immunocytochemistry of activated β-catenin (ABC) and phosphorylated JNK (pJNK) suggests canonical pathway activity already on day 1, but noncanonical pathway activity mainly on day 2 during mesoderm development. Scale bar, 100 μm.

(H) qPCR analysis showing expression of the canonical Wnt target gene *AXIN2* and of *WNT3* already on day 1, in the presence of canonical Wnt activation (CHIR99021) as shown in (E).

(I) qPCR analysis showing expression of *WNT5A* and *5B* mainly on day 2 during mesoderm development, in the presence of canonical Wnt activation (CHIR99021) as shown in (E).

qPCR data are presented as means ± SEM for n = 3 independent experiments. *GAPDH* was used as a housekeeping gene. Asterisk indicates a statistically significant difference between experimental and control samples detected using one-way ANOVA (*$p < 0.05$) for (C) and (D) and two-way ANOVA for (F).

*$p < 0.05$; **$p < 0.01$; ***$p < 0.001$; ****$p < 0.0001$.

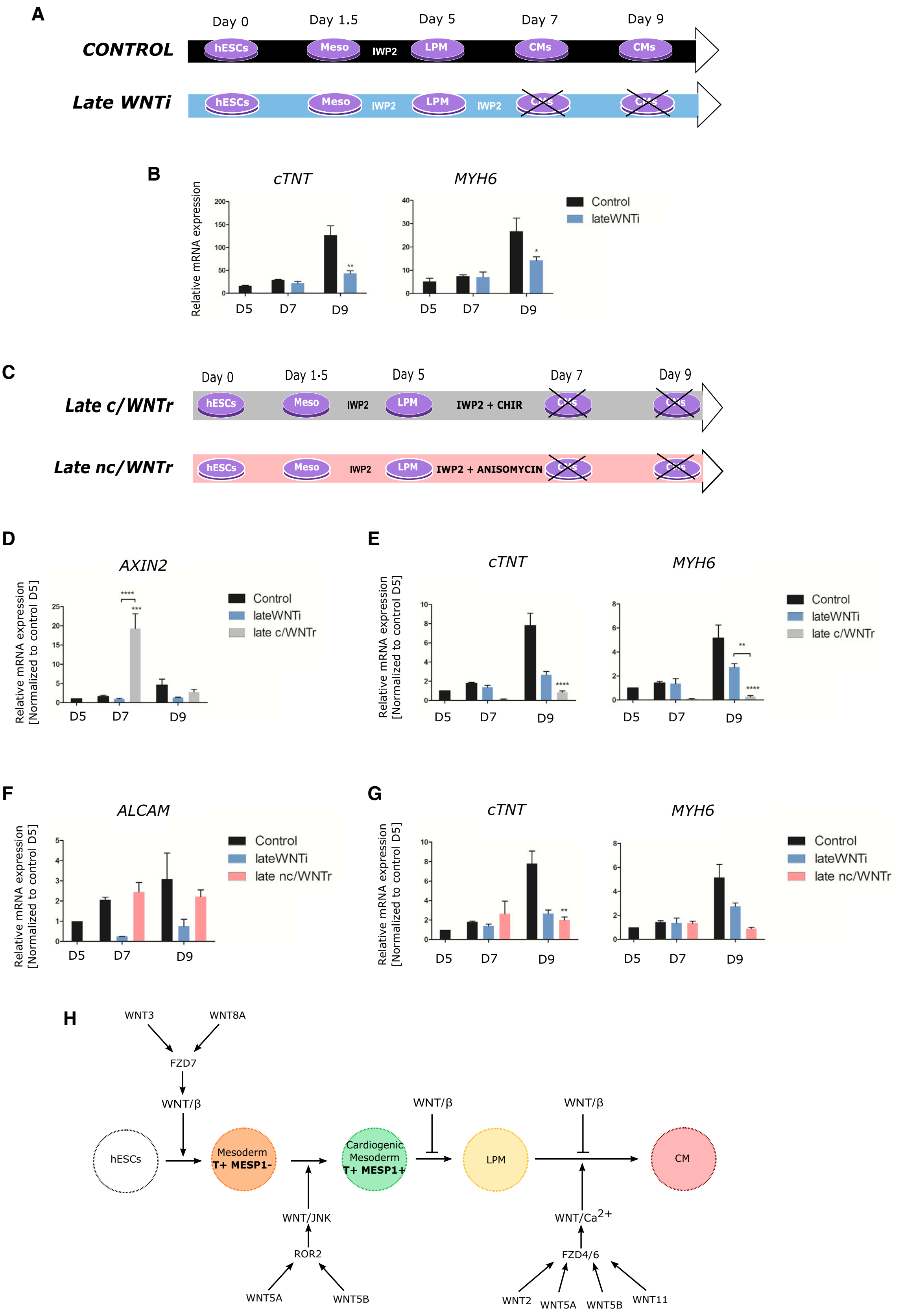

(legend on next page)

DISCUSSION

Vertebrate and invertebrate models have proved fundamental for gaining an understanding of animal heart development and the signaling and transcriptional network mechanisms governing this process. However, the molecular mechanisms underlying human heart development are still largely unclear. hESCs offer unprecedented opportunities to model and study human heart development in vitro. However, so far most of the effort in the field has been directed toward designing efficient protocols to differentiate human cardiomyocytes in vitro. Interestingly, a vast majority of these protocols relies on experimental manipulation of Wnt signaling mechanisms (e.g., Bauwens et al., 2011; Burridge et al., 2014; Chen et al., 2012; Elliott et al., 2011; Gonzalez et al., 2011; Hemmi et al., 2014; Karakikes et al., 2014; Kattman et al., 2011; Kempf et al., 2014; Lian et al., 2012; Otsuji et al., 2014; Paige et al., 2010; Phillips et al., 2008; Ting et al., 2014; Yang et al., 2008; Zhang et al., 2015), suggesting important roles for WNT signaling also in human heart development. However, while Wnt signaling clearly represents a key regulator of vertebrate heart development and particularly of cardiomyocyte differentiation (reviewed by Hoppler et al., 2014), at present there is no detailed understanding of the players and the fundamental roles of Wnt signaling at sequential developmental stages leading to human embryonic cardiomyocyte differentiation. We therefore specifically set out to test whether knowledge from animal model systems would be confirmed for human cardiomyocyte development. Using established hESC differentiation protocols, we studied the activity and requirement of Wnt signaling pathways and identified *WNT* signal and receptor genes during human cardiomyocyte differentiation.

Evidence from model systems had previously suggested a requirement for Wnt signaling during mesoderm induction. In fact, Brachyury is a known target of β-catenin-mediated Wnt signaling (e.g., Arnold et al., 2000; Mendjan et al., 2014; Yamaguchi et al., 1999; Zhang et al., 2013), while WNT/JNK signaling had previously been associated with morphogenesis at gastrulation (Hardy et al., 2008; Tada et al., 2002; Tada and Kai, 2009). Here, we not only confirm that similarly hESCs can only efficiently differentiate in vitro into mesoderm when Wnt signaling is active, but also show that canonical Wnt signaling acts before noncanonical Wnt signaling in humans (Figure 4H). Our data suggest that WNT3 and WNT8A regulate *BRY* (*T*) expression and mesoderm induction via the canonical pathway, after which WNT5A and WNT5B activate JNK-mediated pathway activity to regulate *MESP1* expression, thereby indicating a role for WNT/JNK signaling that goes beyond regulating morphogenesis during gastrulation in the intact embryo. Consistently, our results also show expression of *FZD7* and the noncanonical receptor *ROR2* during mesoderm induction. Interestingly *FZD7* had previously been identified in pluripotent hESCs, where it plays a role in canonical WNT3-mediated self-renewal (Fernandez et al., 2014). Our results suggest that WNT3 and WNT8A and the canonical pathway regulate mesoderm induction via the FZD7 receptor (although additional roles for FZD7 in mediating noncanonical mechanisms cannot be ruled out, e.g., Medina et al., 2000), while ROR2 mediates

Figure 4. Wnt Signaling Function during Cardiomyocyte Differentiation

(A) Schematic description of the protocol to study the requirement for Wnt signaling pathway function during cardiomyocyte differentiation. The protocol AB + WNTi (as in Figure 1A) is modified to extend the inhibition of Wnt signaling mechanisms (by addition of IWP2) until day 7 (Late WNTi). AB + WNTi protocol is used as a control, as indicated.

(B) Gene-expression analysis (qPCR) of cardiomyocyte markers with and without extended Wnt inhibition, as in (A). Note the reduced cardiomyocyte marker gene expression with extended Wnt inhibition.

(C) Schematic description of the protocol to study whether Wnt signaling requirement can be rescued by reinstating canonical Wnt/β-catenin signaling (with CHIR99021, Late c/WNTr) or noncanonical Wnt/JNK signaling (with anisomycin, Late nc/WNTr).

(D) Gene-expression analysis (qPCR) of the canonical Wnt target gene *AXIN2*. Note strong induction of *AXIN2* expression by addition of CHIR99021.

(E) Gene-expression analysis (qPCR) of cardiomyocyte markers in the different conditions shown in (C). Note that reinstating canonical Wnt/β-catenin signaling further reduces cardiomyocyte marker gene expression.

(F) Gene-expression analysis (qPCR) of the proposed noncanonical Wnt target gene *ALCAM* in the different conditions shown in (C). Note strong induction of *ALCAM* expression by addition of anisomycin.

(G) Gene-expression analysis (qPCR) of cardiomyocyte markers in the different conditions shown in (C). Note that reinstating noncanonical Wnt/JNK signaling fails to rescue cardiomyocyte marker gene expression.

(H) Schematic description of conclusions. Note that the function of canonical and noncanonical Wnt signaling early during mesoderm development can be separated temporally and in terms of marker gene induction. Further note that later canonical Wnt signaling is still inhibitory, while noncanonical Wnt signaling is required for cardiomyocyte differentiation (see text for further details).

qPCR data are presented as means ± SEM for n = 3 independent experiments. *GAPDH* was used as a housekeeping gene. Asterisk indicates a statistically significant difference between samples as indicated, which was detected using two-way ANOVA.

*$p < 0.05$; **$p < 0.01$; ***$p < 0.001$; ****$p < 0.0001$.

WNT5A/B role in the commitment of the earliest cardiogenic mesoderm (i.e., MESP1-positive cells).

We further confirm in human cells that inhibition of specifically canonical Wnt signaling following mesoderm induction is both necessary and sufficient for subsequent efficient cardiomyocyte differentiation (Figure 4H). Experimental activation of noncanonical Wnt/JNK signaling has been shown in a variety of experimental models to promote cardiac specification (Bisson et al., 2015; Eisenberg and Eisenberg, 1999; Terami et al., 2004), which, it has been argued, may function by antagonizing canonical pathway activity (reviewed in Hoppler et al., 2014). However, we observe little activation of noncanonical Wnt/JNK signaling following mesoderm induction and little evidence of pathway-relevant Wnt ligand or Wnt receptor gene expression, which suggests that noncanonical Wnt/JNK signaling may not be required at this specific stage. Consistently, we find that experimental inhibition of JNK signaling alone at this stage does not affect differentiation of mesodermal cells into LPM and subsequently into cardiomyocytes. The effects observed by others on cardiac specification after experimental activation of noncanonical Wnt/JNK signaling may be related to the earlier function of Wnt/JNK signaling we have discovered (see above).

Finally, until now nothing was known about the role of Wnt signaling during differentiation from cardiac precursors into human cardiomyocytes. In animal model systems, a prominent role of noncanonical Wnt/JNK signaling at this stage has been suggested, while it is still not clear whether canonical Wnt signaling may play further roles following cardiac mesoderm induction for subsequent cardiomyocyte differentiation (both reviewed in Gessert and Kühl, 2010). We observe sustained expression of presumably noncanonical *WNT5A*, *WNT5B*, and *WNT11* at this stage (Figure 4H), but also *WNT2*, which was conventionally believed to activate β-catenin-mediated signaling (Goss et al., 2009). When Wnt signaling was inhibited at this late stage, cardiomyocyte differentiation was negatively affected. However, activation of canonical Wnt signaling at this stage was found to be detrimental for terminal cardiomyocyte differentiation, suggesting a role solely for noncanonical Wnt signaling during cardiomyocyte differentiation. The involvement of *wnt2* in cardiomyocyte differentiation from ESCs mediated by noncanonical Wnt signaling had previously been identified in the mouse (Onizuka et al., 2012), which our results now suggest are conserved in human development. Our results further suggest that noncanonical signaling at this stage is mediated by *FZD4* (Abdul-Ghani et al., 2011; Descamps et al., 2012) and *FZD6* (Gray et al., 2011; Guo et al., 2004) receptors. However, experimental activation of JNK signaling alone proved insufficient for rescuing cardiomyocyte differentiation. Therefore, future experiments will be required to explore the role of other noncanonical Wnt signaling pathways in late cardiomyocyte differentiation, which may work together with or instead of the JNK-mediated pathway. Given that Ca^{2+} is needed for proper cardiac development and heart function, the Wnt/Ca^{2+} pathway may represent a mediator of noncanonical Wnt signaling in this context (Panakova et al., 2010).

Overall, our study demonstrates that canonical and noncanonical Wnt pathway mechanisms have specific roles in regulating human cardiomyocyte differentiation that go beyond simple mutual antagonism. The established hESC differentiation protocols we used clearly proved useful to distinguish different phases of Wnt pathway activity and requirement, as well as stage-specific Wnt signal and Wnt receptor gene expression (Figure 4H). Future analysis may be able to determine more precisely whether canonical and noncanonical Wnt signaling mechanisms operate at exactly the same stage and in exactly the same tissue, and whether specific Wnt ligands and receptors mediate canonical and others noncanonical pathway activation. Given the outstanding impact of cardiovascular disease on society, a more detailed understanding of the regulatory mechanisms underlying human embryonic cardiac muscle commitment and differentiation will provide fundamental insights into the etiology of congenital heart defects, and may also suggest innovative therapeutic applications in the field of regenerative medicine.

EXPERIMENTAL PROCEDURES

hESC Differentiation Protocols

AB + WNTi

hESCs (NSCB no. WA09 [H9] and WA07 [H7]) were grown on inactivated mouse embryonic fibroblasts, as previously described (Thomson et al., 1998). All experiments were repeated with both cell lines with comparable results, but results presented here are from H9 culture. Differentiation toward mesoderm, LPM, and beating cardiomyocytes was performed in chemically defined medium containing polyvinyl alcohol, essentially as previously described (Bernardo et al., 2011; Mendjan et al., 2014) with only minor modifications (Figure 1A): for the first 36 hr of the protocol cells were stimulated with 20 ng/mL fibroblast growth factor 2 (FGF2),10 μM phosphoinositide 3-kinase inhibitor (LY294002), 20 ng/mL activin A, and 10 ng/mL BMP4, for mesoderm induction; addition of 20 ng/mL FGF2, 50 ng/mL BMP4, 0.5 μM retinoic acid, and 5 μM of Wnt inhibitor (either IWR1 or IWP2) from day 1.5 to day 5 allowed LPM differentiation; finally, treatment with 5 ng/mL FGF2 and 10 ng/mL BMP4 allowed differentiation into cardiomyocytes.

C + WNTi

hESCs (NSCB no. WA09 [H9] and WA07 [H7]) were grown on mTeSR1/Matrigel Platform as per the manufacturer's instructions. All experiments were repeated with both cell lines with comparable results, but results presented here are from H7 culture. Differentiation toward mesoderm, LPM, and beating cardiomyocytes

was performed essentially as previously described (Lian et al., 2013) with only minor modifications (Figure 1B): for the first 48 hr of the protocol cells were stimulated with 12 μM CHIR99021 in RPMI/low insulin-B27 for mesoderm induction; subsequently, addition of the Wnt inhibitor IWP2 (5 μM) from days 2 to 4 in RPMI/low insulin-B27 allowed LPM differentiation; cells were then grown in RPMI/low insulin-B27 for a further 2 days; finally, medium was changed to RPMI/B27 on day 6 and cells were kept in culture until day 9 to allow differentiation into cardiomyocytes.

SUPPLEMENTAL INFORMATION

Supplemental Information includes Supplemental Experimental Procedures, two figures, three tables, and two movies and can be found with this article online at http://dx.doi.org/10.1016/j.stemcr.2016.08.008.

AUTHOR CONTRIBUTIONS

S.M. carried out the experiments and analysis, helped in experimental design, and wrote the manuscript. C.N. contributed to design, carried out experiments, and advised on analysis and manuscript. R.J.B. carried out experiments. A.S.B. advised on design, methods, and manuscript, and carried out experiments. K.D. advised on design and manuscript. S.H. designed the study and wrote the manuscript.

ACKNOWLEDGMENTS

Our thanks go to Gioia Polidori Francisco for training and discussions, Kate Watt and Yvonne Turnbull for technical and laboratory managerial support, Kadri Oras and Laura Ferguson for experimental support, Po-Lin So and Bruce Conklin (Gladstone Institutes) for providing their unpublished protocols, and Yukio Nakamura for discussion. This research is supported by the British Heart Foundation (PG/12/75/29851) and the Institute of Medical Sciences. A.S.B. was supported by the British Heart Foundation (FS/12/37/29516).

Received: September 24, 2015
Revised: August 11, 2016
Accepted: August 11, 2016
Published: September 15, 2016

REFERENCES

Abdul-Ghani, M., Dufort, D., Stiles, R., De Repentigny, Y., Kothary, R., and Megeney, L.A. (2011). Wnt11 promotes cardiomyocyte development by caspase-mediated suppression of canonical Wnt signals. Mol. Cell Biol. *31*, 163–178.

Arnold, S.J., Stappert, J., Bauer, A., Kispert, A., Herrmann, B.G., and Kemler, R. (2000). Brachyury is a target gene of the Wnt/beta-catenin signaling pathway. Mech. Dev. *91*, 249–258.

Bauwens, C.L., Song, H., Thavandiran, N., Ungrin, M., Masse, S., Nanthakumar, K., Seguin, C., and Zandstra, P.W. (2011). Geometric control of cardiomyogenic induction in human pluripotent stem cells. Tissue Eng. Part A *17*, 1901–1909.

Bernardo, A.S., Faial, T., Gardner, L., Niakan, K.K., Ortmann, D., Senner, C.E., Callery, E.M., Trotter, M.W., Hemberger, M., Smith, J.C., et al. (2011). BRACHYURY and CDX2 mediate BMP-induced differentiation of human and mouse pluripotent stem cells into embryonic and extraembryonic lineages. Cell Stem Cell *9*, 144–155.

Bisson, J.A., Mills, B., Paul Helt, J.C., Zwaka, T.P., and Cohen, E.D. (2015). Wnt5a and Wnt11 inhibit the canonical Wnt pathway and promote cardiac progenitor development via the Caspase-dependent degradation of AKT. Dev. Biol. *398*, 80–96.

Burridge, P.W., Matsa, E., Shukla, P., Lin, Z.C., Churko, J.M., Ebert, A.D., Lan, F., Diecke, S., Huber, B., Mordwinkin, N.M., et al. (2014). Chemically defined generation of human cardiomyocytes. Nat. Methods *11*, 855–860.

Cao, N., Liang, H., Huang, J., Wang, J., Chen, Y., Chen, Z., and Yang, H.T. (2013). Highly efficient induction and long-term maintenance of multipotent cardiovascular progenitors from human pluripotent stem cells under defined conditions. Cell Res. *23*, 1119–1132.

Chen, B., Dodge, M.E., Tang, W., Lu, J., Ma, Z., Fan, C.W., Wei, S., Hao, W., Kilgore, J., Williams, N.S., et al. (2009). Small molecule-mediated disruption of Wnt-dependent signaling in tissue regeneration and cancer. Nat. Chem. Biol. *5*, 100–107.

Chen, V.C., Couture, S.M., Ye, J., Lin, Z., Hua, G., Huang, H.I., Wu, J., Hsu, D., Carpenter, M.K., and Couture, L.A. (2012). Scalable GMP compliant suspension culture system for human ES cells. Stem Cell Res. *8*, 388–402.

Choudhry, P., and Trede, N.S. (2013). DiGeorge syndrome gene tbx1 functions through wnt11r to regulate heart looping and differentiation. PLoS One *8*, e58145.

Cizelsky, W., Tata, A., Kuhl, M., and Kuhl, S.J. (2014). The Wnt/JNK signaling target gene alcam is required for embryonic kidney development. Development *141*, 2064–2074.

Cohen, E.D., Miller, M.F., Wang, Z., Moon, R.T., and Morrisey, E.E. (2012). Wnt5a and Wnt11 are essential for second heart field progenitor development. Development *139*, 1931–1940.

David, R., Brenner, C., Stieber, J., Schwarz, F., Brunner, S., Vollmer, M., Mentele, E., Müller-Höcker, J., Kitajima, S., Lickert, H., et al. (2008). MesP1 drives vertebrate cardiovascular differentiation through Dkk-1-mediated blockade of Wnt-signalling. Nat. Cell Biol. *10*, 338–345.

Descamps, B., Sewduth, R., Ferreira Tojais, N., Jaspard, B., Reynaud, A., Sohet, F., Lacolley, P., Allieres, C., Lamaziere, J.M., Moreau, C., et al. (2012). Frizzled 4 regulates arterial network organization through noncanonical Wnt/planar cell polarity signaling. Circ. Res. *110*, 47–58.

Eisenberg, C.A., and Eisenberg, L.M. (1999). WNT11 promotes cardiac tissue formation of early mesoderm. Dev. Dyn. *216*, 45–58.

Elliott, D.A., Braam, S.R., Koutsis, K., Ng, E.S., Jenny, R., Lagerqvist, E.L., Biben, C., Hatzistavrou, T., Hirst, C.E., Yu, Q.C., et al. (2011). NKX2-5(eGFP/w) hESCs for isolation of human cardiac progenitors and cardiomyocytes. Nat. Methods *8*, 1037–1040.

Fernandez, A., Huggins, I.J., Perna, L., Brafman, D., Lu, D., Yao, S., Gaasterland, T., Carson, D.A., and Willert, K. (2014). The WNT receptor FZD7 is required for maintenance of the pluripotent state in

human embryonic stem cells. Proc. Natl. Acad. Sci. USA *111*, 1409–1414.

Gessert, S., and Kühl, M. (2010). The multiple phases and faces of wnt signaling during cardiac differentiation and development. Circ. Res. *107*, 186–199.

Gessert, S., Maurus, D., Brade, T., Walther, P., Pandur, P., and Kühl, M. (2008). DM-GRASP/ALCAM/CD166 is required for cardiac morphogenesis and maintenance of cardiac identity in first heart field derived cells. Dev. Biol. *321*, 150–161.

Gonzalez, R., Lee, J.W., and Schultz, P.G. (2011). Stepwise chemically induced cardiomyocyte specification of human embryonic stem cells. Angew. Chem. Int. Ed. Engl. *50*, 11181–11185.

Goss, A.M., Tian, Y., Tsukiyama, T., Cohen, E.D., Zhou, D., Lu, M.M., Yamaguchi, T.P., and Morrisey, E.E. (2009). Wnt2/2b and beta-catenin signaling are necessary and sufficient to specify lung progenitors in the foregut. Dev. Cell *17*, 290–298.

Gray, R.S., Roszko, I., and Solnica-Krezel, L. (2011). Planar cell polarity: coordinating morphogenetic cell behaviors with embryonic polarity. Dev. Cell *21*, 120–133.

Guo, N., Hawkins, C., and Nathans, J. (2004). Frizzled6 controls hair patterning in mice. Proc. Natl. Acad. Sci. USA *101*, 9277–9281.

Hardy, K.M., Garriock, R.J., Yatskievych, T.A., D'Agostino, S.L., Antin, P.B., and Krieg, P.A. (2008). Non-canonical Wnt signaling through Wnt5a/b and a novel Wnt11 gene, Wnt11b, regulates cell migration during avian gastrulation. Dev. Biol. *320*, 391–401.

He, X., Saint-Jeannet, J.P., Wang, Y., Nathans, J., Dawid, I., and Varmus, H. (1997). A member of the Frizzled protein family mediating axis induction by Wnt-5A. Science *275*, 1652–1654.

Hemmi, N., Tohyama, S., Nakajima, K., Kanazawa, H., Suzuki, T., Hattori, F., Seki, T., Kishino, Y., Hirano, A., Okada, M., et al. (2014). A massive suspension culture system with metabolic purification for human pluripotent stem cell-derived cardiomyocytes. Stem Cells Transl. Med. *3*, 1473–1483.

Hoppler, S., and Nakamura, Y. (2014). Cell-to-cell signalling in development: Wnt signalling. eLS. http://dx.doi.org/10.1002/9780470015902.a0002331.pub2.

Hoppler, S., Mazzotta, S., and Kühl, M. (2014). Wnt signaling in heart organogenesis. In Wnt Signaling in Development and Disease, S.P. Hoppler and R.T. Moon, eds. (John Wiley), pp. 293–301.

Karakikes, I., Senyei, G.D., Hansen, J., Kong, C.W., Azeloglu, E.U., Stillitano, F., Lieu, D.K., Wang, J., Ren, L., Hulot, J.S., et al. (2014). Small molecule-mediated directed differentiation of human embryonic stem cells toward ventricular cardiomyocytes. Stem Cells Transl. Med. *3*, 18–31.

Kattman, S.J., Witty, A.D., Gagliardi, M., Dubois, N.C., Niapour, M., Hotta, A., Ellis, J., and Keller, G. (2011). Stage-specific optimization of activin/nodal and BMP signaling promotes cardiac differentiation of mouse and human pluripotent stem cell lines. Cell Stem Cell *8*, 228–240.

Kempf, H., Olmer, R., Kropp, C., Ruckert, M., Jara-Avaca, M., Robles-Diaz, D., Franke, A., Elliott, D.A., Wojciechowski, D., Fischer, M., et al. (2014). Controlling expansion and cardiomyogenic differentiation of human pluripotent stem cells in scalable suspension culture. Stem Cell Rep. *3*, 1132–1146.

Kilian, B., Mansukoski, H., Barbosa, F.C., Ulrich, F., Tada, M., and Heisenberg, C.P. (2003). The role of Ppt/Wnt5 in regulating cell shape and movement during zebrafish gastrulation. Mech. Dev. *120*, 467–476.

Laflamme, M.A., Chen, K.Y., Naumova, A.V., Muskheli, V., Fugate, J.A., Dupras, S.K., Reinecke, H., Xu, C., Hassanipour, M., Police, S., et al. (2007). Cardiomyocytes derived from human embryonic stem cells in pro-survival factors enhance function of infarcted rat hearts. Nat. Biotechnol. *25*, 1015–1024.

Lavery, D.L., Martin, J., Turnbull, Y.D., and Hoppler, S. (2008). Wnt6 signaling regulates heart muscle development during organogenesis. Dev. Biol. *323*, 177–188.

Lian, X., Hsiao, C., Wilson, G., Zhu, K., Hazeltine, L.B., Azarin, S.M., Raval, K.K., Zhang, J., Kamp, T.J., and Palecek, S.P. (2012). Robust cardiomyocyte differentiation from human pluripotent stem cells via temporal modulation of canonical Wnt signaling. Proc. Natl. Acad. Sci. USA *109*, E1848–E1857.

Lian, X., Zhang, J., Azarin, S.M., Zhu, K., Hazeltine, L.B., Bao, X., Hsiao, C., Kamp, T.J., and Palecek, S.P. (2013). Directed cardiomyocyte differentiation from human pluripotent stem cells by modulating Wnt/beta-catenin signaling under fully defined conditions. Nat. Protoc. *8*, 162–175.

Medina, A., Reintsch, W., and Steinbeisser, H. (2000). Xenopus frizzled 7 can act in canonical and non-canonical Wnt signaling pathways: implications on early patterning and morphogenesis. Mech. Dev. *92*, 227–237.

Mendjan, S., Mascetti, V.L., Ortmann, D., Ortiz, M., Karjosukarso, D.W., Ng, Y., Moreau, T., and Pedersen, R.A. (2014). Nanog and CDX2 pattern distinct subtypes of human mesoderm during exit from pluripotency. Cell Stem Cell *15*, 310–325.

Naito, A.T., Shiojima, I., Akazawa, H., Hidaka, K., Morisaki, T., Kikuchi, A., and Komuro, I. (2006). Developmental stage-specific biphasic roles of Wnt/beta-catenin signaling in cardiomyogenesis and hematopoiesis. Proc. Natl. Acad. Sci. USA *103*, 19812–19817.

Onizuka, T., Yuasa, S., Kusumoto, D., Shimoji, K., Egashira, T., Ohno, Y., Kageyama, T., Tanaka, T., Hattori, F., Fujita, J., et al. (2012). Wnt2 accelerates cardiac myocyte differentiation from ES-cell derived mesodermal cells via non-canonical pathway. J. Mol. Cell Cardiol. *52*, 650–659.

Otsuji, T.G., Bin, J., Yoshimura, A., Tomura, M., Tateyama, D., Minami, I., Yoshikawa, Y., Aiba, K., Heuser, J.E., Nishino, T., et al. (2014). A 3D sphere culture system containing functional polymers for large-scale human pluripotent stem cell production. Stem Cell Rep. *2*, 734–745.

Paige, S.L., Osugi, T., Afanasiev, O.K., Pabon, L., Reinecke, H., and Murry, C.E. (2010). Endogenous Wnt/beta-catenin signaling is required for cardiac differentiation in human embryonic stem cells. PLoS One *5*, e11134.

Panakova, D., Werdich, A.A., and Macrae, C.A. (2010). Wnt11 patterns a myocardial electrical gradient through regulation of the L-type Ca(2+) channel. Nature *466*, 874–878.

Pandur, P., Lasche, M., Eisenberg, L.M., and Kuhl, M. (2002a). Wnt-11 activation of a non-canonical Wnt signalling pathway is required for cardiogenesis. Nature *418*, 636–641.

Pandur, P., Maurus, D., and Kuhl, M. (2002b). Increasingly complex: new players enter the Wnt signaling network. Bioessays *24*, 881–884.

Phillips, B.W., Horne, R., Lay, T.S., Rust, W.L., Teck, T.T., and Crook, J.M. (2008). Attachment and growth of human embryonic stem cells on microcarriers. J. Biotechnol. *138*, 24–32.

Rosenthal, N., and Harvey, R.P. (2010). Heart Development and Regeneration, *vol. 2* (Academic Press).

Singh, A.M., Li, F.Q., Hamazaki, T., Kasahara, H., Takemaru, K., and Terada, N. (2007). Chibby, an antagonist of the Wnt/beta-catenin pathway, facilitates cardiomyocyte differentiation of murine embryonic stem cells. Circulation *115*, 617–626.

Staal, F.J.T., van Noort, M., Strous, G.J., and Clevers, H.C. (2002). Wnt signals are transmitted through N-terminally dephosphorylated beta-catenin. EMBO Rep. *3*, 63–68.

Sylva, M., van den Hoff, M.J., and Moorman, A.F. (2014). Development of the human heart. Am. J. Med. Genet. A *164A*, 1347–1371.

Tada, M., and Kai, M. (2009). Noncanonical Wnt/PCP signaling during vertebrate gastrulation. Zebrafish *6*, 29–40.

Tada, M., Concha, M.L., and Heisenberg, C.P. (2002). Non-canonical Wnt signalling and regulation of gastrulation movements. Semin. Cell Dev. Biol. *13*, 251–260.

Terami, H., Hidaka, K., Katsumata, T., Iio, A., and Morisaki, T. (2004). Wnt11 facilitates embryonic stem cell differentiation to Nkx2.5-positive cardiomyocytes. Biochem. Biophys. Res. Commun. *325*, 968–975.

Thomson, J.A., Itskovitz-Eldor, J., Shapiro, S.S., Waknitz, M.A., Swiergiel, J.J., Marshall, V.S., and Jones, J.M. (1998). Embryonic stem cell lines derived from human blastocysts. Science *282*, 1145–1147.

Ting, S., Chen, A., Reuveny, S., and Oh, S. (2014). An intermittent rocking platform for integrated expansion and differentiation of human pluripotent stem cells to cardiomyocytes in suspended microcarrier cultures. Stem Cell Res. *13*, 202–213.

Ueno, S., Weidinger, G., Osugi, T., Kohn, A.D., Golob, J.L., Pabon, L., Reinecke, H., Moon, R.T., and Murry, C.E. (2007). Biphasic role for Wnt/beta-catenin signaling in cardiac specification in zebrafish and embryonic stem cells. Proc. Natl. Acad. Sci. USA *104*, 9685–9690.

Ulrich, F., Krieg, M., Schotz, E.M., Link, V., Castanon, I., Schnabel, V., Taubenberger, A., Mueller, D., Puech, P.H., and Heisenberg, C.P. (2005). Wnt11 functions in gastrulation by controlling cell cohesion through Rab5c and E-cadherin. Dev. Cell *9*, 555–564.

Willems, E., Spiering, S., Davidovics, H., Lanier, M., Xia, Z., Dawson, M., Cashman, J., and Mercola, M. (2011). Small-molecule inhibitors of the Wnt pathway potently promote cardiomyocytes from human embryonic stem cell-derived mesoderm. Circ. Res. *109*, 360–364.

Yamaguchi, T.P. (2008). Genetics of Wnt signaling during early mammalian development. Methods Mol. Biol. *468*, 287–305.

Yamaguchi, T.P., Takada, S., Yoshikawa, Y., Wu, N., and McMahon, A.P. (1999). T (Brachyury) is a direct target of Wnt3a during paraxial mesoderm specification. Genes Dev. *13*, 3185–3190.

Yan, D., Wiesmann, M., Rohan, M., Chan, V., Jefferson, A.B., Guo, L., Sakamoto, D., Caothien, R.H., Fuller, J.H., Reinhard, C., et al. (2001). Elevated expression of axin2 and hnkd mRNA provides evidence that Wnt/beta-catenin signaling is activated in human colon tumors. Proc. Natl. Acad. Sci. USA *98*, 14973–14978.

Yang, L., Soonpaa, M.H., Adler, E.D., Roepke, T.K., Kattman, S.J., Kennedy, M., Henckaerts, E., Bonham, K., Abbott, G.W., Linden, R.M., et al. (2008). Human cardiovascular progenitor cells develop from a KDR+ embryonic-stem-cell-derived population. Nature *453*, 524–528.

Zhang, X., Peterson, K.A., Liu, X.S., McMahon, A.P., and Ohba, S. (2013). Gene regulatory networks mediating canonical Wnt signal-directed control of pluripotency and differentiation in embryo stem cells. Stem Cells *31*, 2667–2679.

Zhang, M., Schulte, J.S., Heinick, A., Piccini, I., Rao, J., Quaranta, R., Zeuschner, D., Malan, D., Kim, K.P., Ropke, A., et al. (2015). Universal cardiac induction of human pluripotent stem cells in two and three-dimensional formats: implications for in vitro maturation. Stem Cells *33*, 1456–1469.

Stem Cell Reports, Volume 7

Supplemental Information

Distinctive Roles of Canonical and Noncanonical Wnt Signaling in Human Embryonic Cardiomyocyte Development

Silvia Mazzotta, Carlos Neves, Rory J. Bonner, Andreia S. Bernardo, Kevin Docherty, and Stefan Hoppler

SUPPLEMENTAL INFORMATION

SUPPLEMENTAL FIGURES

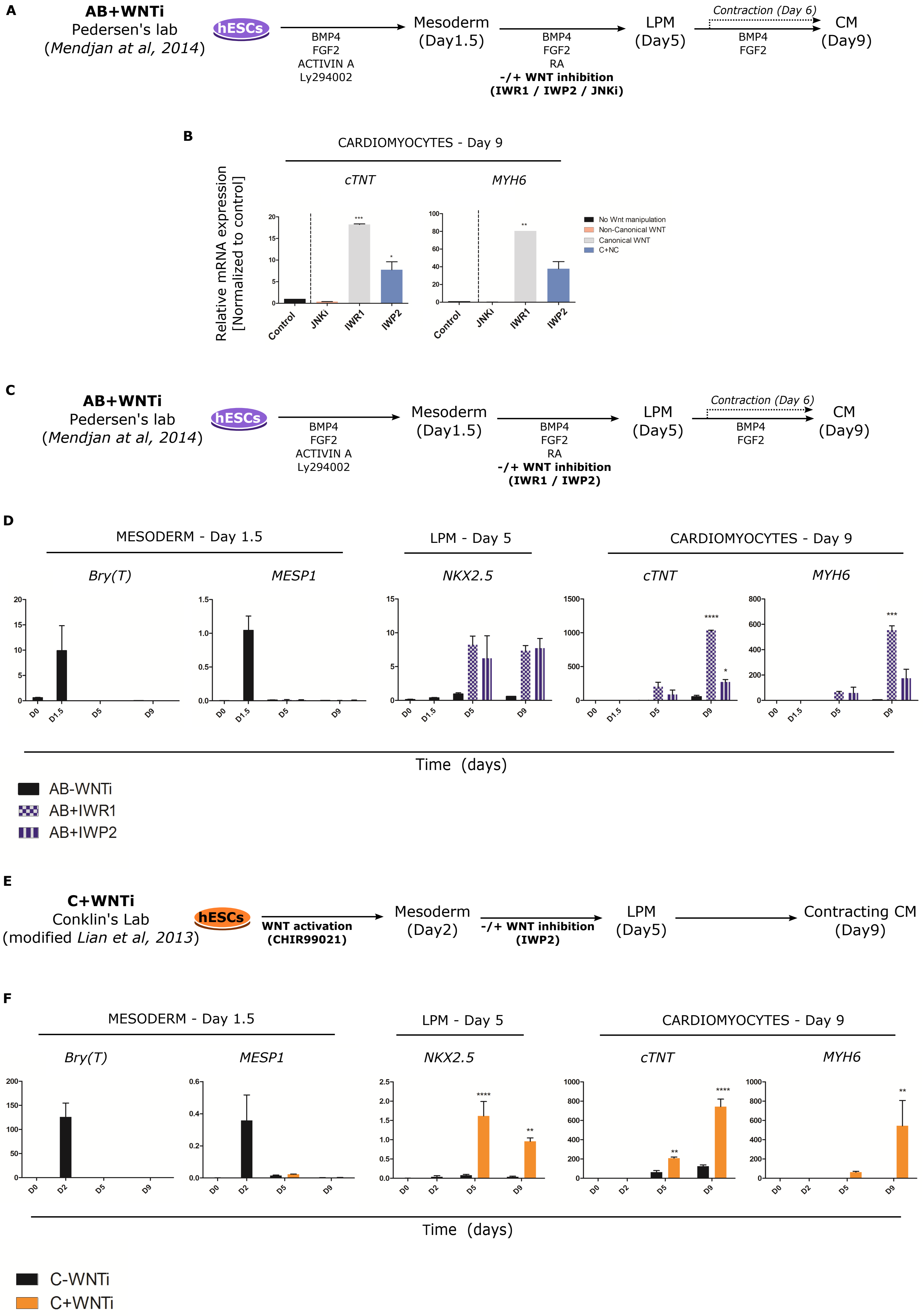
A
AB+WNTi
Pedersen's lab
(Mendjan at al, 2014)
hESCs
BMP4
FGF2
ACTIVIN A
Ly294002
Mesoderm
(Day1.5)
BMP4
FGF2
RA
-/+ WNT inhibition
(IWR1 / IWP2 / JNKi)
LPM
(Day5)
Contraction (Day 6)
BMP4
FGF2
CM
(Day9)
B
CARDIOMYOCYTES - Day 9
cTNT
MYH6
Relative mRNA expression
[Normalized to control]
No Wnt manipulation
Non-Canonical WNT
Canonical WNT
C+NC
Control
JNKi
IWR1
IWP2
C
AB+WNTi
Pedersen's lab
(Mendjan at al, 2014)
hESCs
BMP4
FGF2
ACTIVIN A
Ly294002
Mesoderm
(Day1.5)
BMP4
FGF2
RA
-/+ WNT inhibition
(IWR1 / IWP2)
LPM
(Day5)
Contraction (Day 6)
BMP4
FGF2
CM
(Day9)
D
MESODERM - Day 1.5
LPM - Day 5
CARDIOMYOCYTES - Day 9
Bry(T)
MESP1
NKX2.5
cTNT
MYH6
Relative mRNA expression
D0
D1.5
D5
D9
Time (days)
AB-WNTi
AB+IWR1
AB+IWP2
E
C+WNTi
Conklin's Lab
(modified Lian et al, 2013)
hESCs
WNT activation
(CHIR99021)
Mesoderm
(Day2)
-/+ WNT inhibition
(IWP2)
LPM
(Day5)
Contracting CM
(Day9)
F
MESODERM - Day 1.5
LPM - Day 5
CARDIOMYOCYTES - Day 9
Bry(T)
MESP1
NKX2.5
cTNT
MYH6
Relative mRNA expression
D0
D2
D5
D9
Time (days)
C-WNTi
C+WNTi

Supplemental Figure S1: Study of Wnt signaling requirement for human cardiomyocyte development using hESC differentiation protocols

(**A**) Schematic description of the cardiomyocyte differentiation protocol AB+WNTi, where WNTi is addition of IWP2, IWR1 or JNKi (SP600125) between Day 1.5 and Day 5. (**B**) Cardiomyocyte gene expression study in qPCR on Day 9 of the Protocol described in **S1A**. Note absence of differentiation when just noncanonical Wnt signaling is inhibited. (**C**) Schematic description of the cardiomyocyte differentiation protocol AB+WNTi. (**D**) Gene expression (qPCR analysis) of mesoderm, LPM and Cardiomyocyte markers in the different conditions shown in **Fig. S1C**. Note that inhibition of canonical Wnt signaling is sufficient and necessary for primary cardiomyocyte differentiation. (**E**) Schematic description of the cardiomyocyte differentiation protocol C+WNTi. (**F**) Gene expression (qPCR analysis) of mesoderm, LPM and Cardiomyocyte markers in the different conditions shown in **Fig. S1E.**

qPCR data are presented as mean ± SEM for n=3 biological replicates. *GAPDH* was used as a housekeeping gene. * indicates a statistically significant difference between experimental and control samples detected using 1-way ANOVA for panel B and 2-way ANOVA for panels D and F. * $P<0.05$; ** $P<0.01$; *** $P<0.001$; **** $P<0.0001$.

A

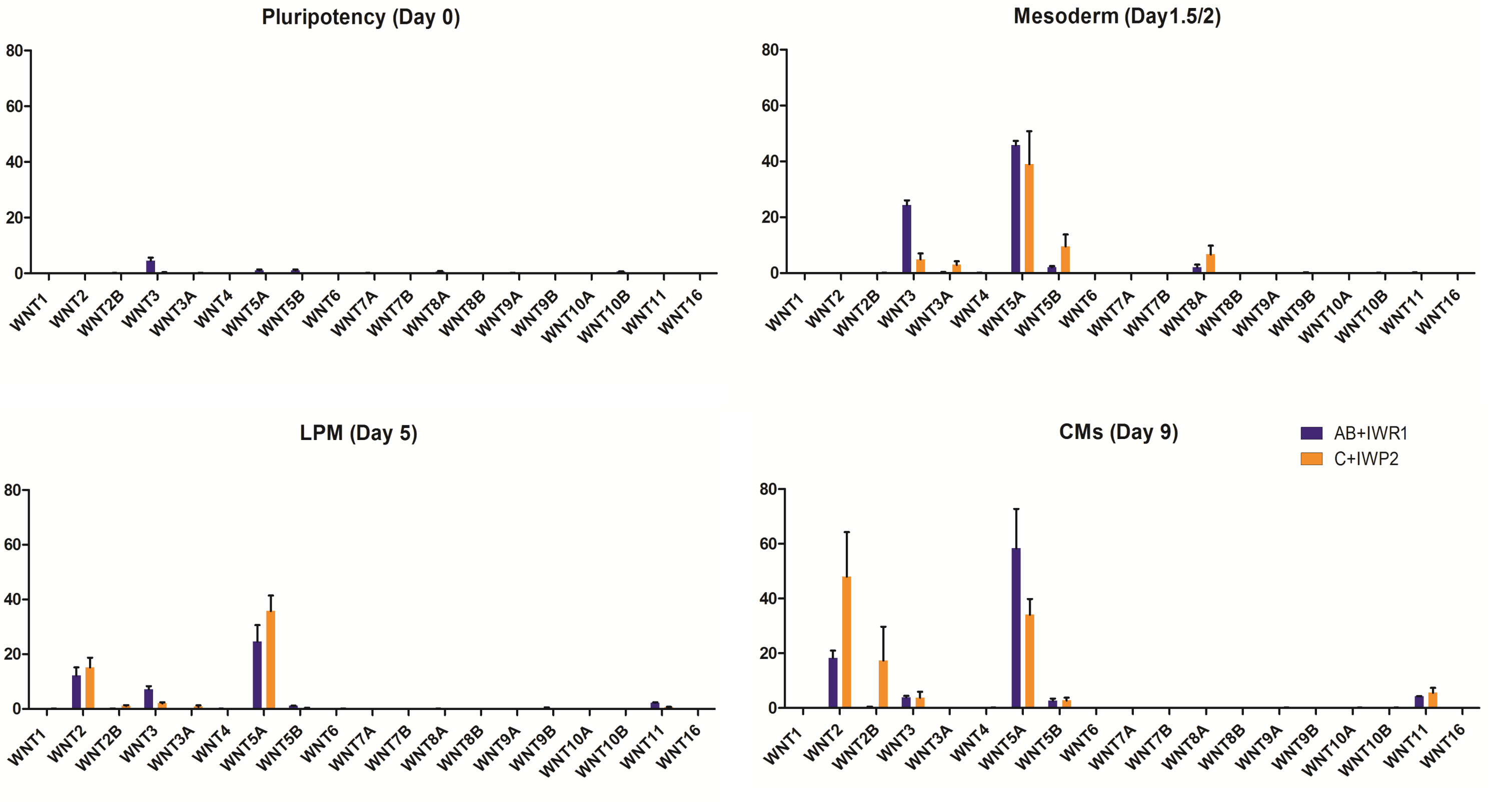
Pluripotency (Day 0)
Mesoderm (Day1.5/2)
LPM (Day 5)
CMs (Day 9)
AB+IWR1
C+IWP2
0
20
40
60
80
WNT1
WNT2
WNT2B
WNT3
WNT3A
WNT4
WNT5A
WNT5B
WNT6
WNT7A
WNT7B
WNT8A
WNT8B
WNT9A
WNT9B
WNT10A
WNT10B
WNT11
WNT16

B

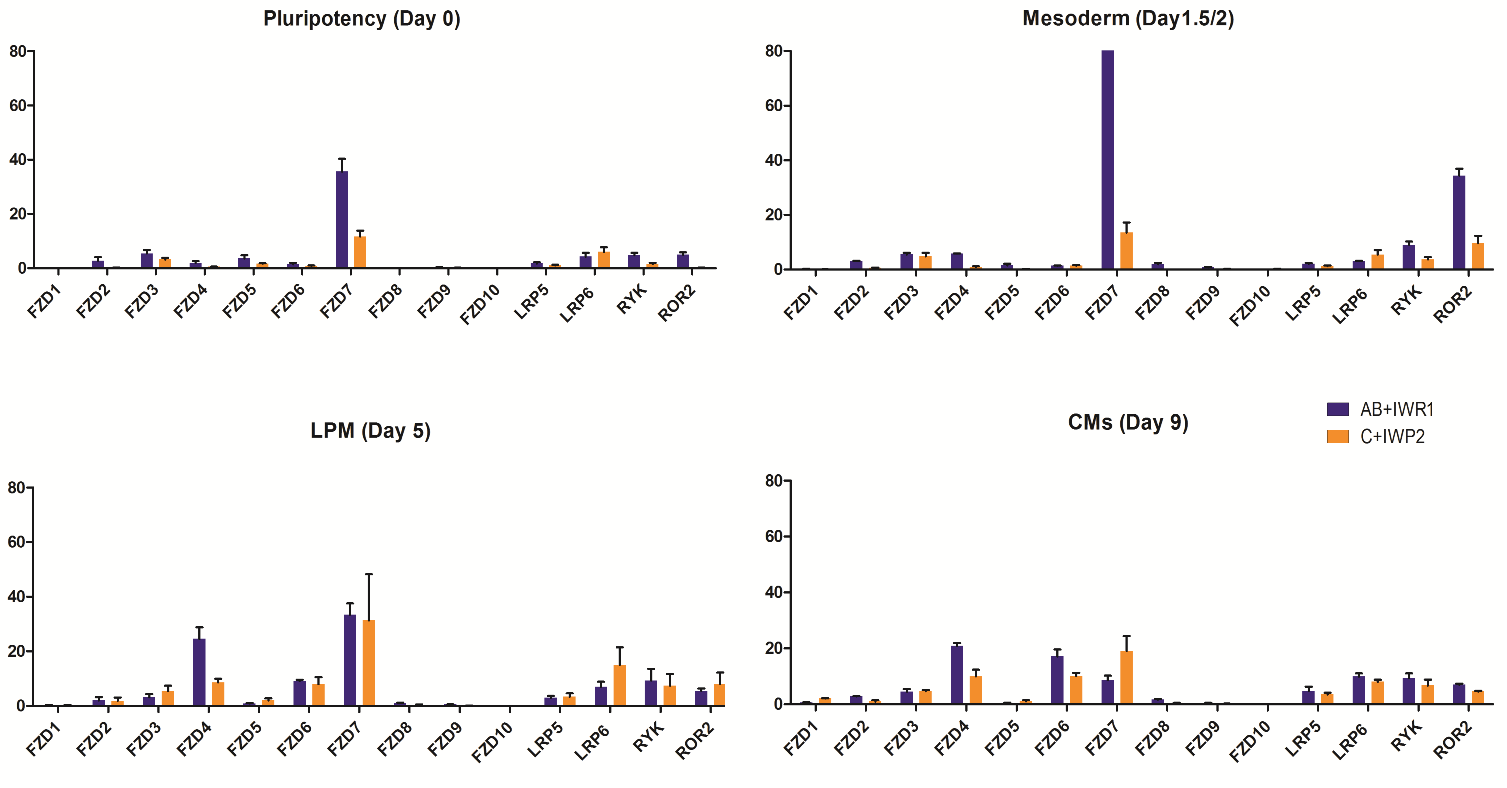
Pluripotency (Day 0)
Mesoderm (Day1.5/2)
LPM (Day 5)
CMs (Day 9)
AB+IWR1
C+IWP2
0
20
40
60
80
FZD1
FZD2
FZD3
FZD4
FZD5
FZD6
FZD7
FZD8
FZD9
FZD10
LRP5
LRP6
RYK
ROR2

Supplemental Figure S2: Wnt signal and receptor gene expression in human cardiomyocyte development using hESC differentiation protocols

(**A**) qPCR profile of all Wnt gene expression in AB+WNTi and C+WNTi as indicated. Data is plot by differentiation stage/day. Note prominent expression of *WNT3, 8A, 5A and 5B* early during mesoderm induction and expression of *WNT5A*, *5B*, *2* and *11* later during cardiomyocyte differentiation. (**B**) qPCR profile of all Wnt receptor and co-receptor gene expression in AB+WNTi and C+WNTi as indicated. Data is plot by differentiation stage/day. Note prominent expression of *FZD7* and ROR2 early during mesoderm induction and *FZD4* and *FZD6* later during cardiomyocyte differentiation.

qPCR data are presented as mean ± SEM for n=3 biological replicates. *GAPDH* was used as a housekeeping gene.

SUPPLEMENTAL EXPERIMENTAL PROCEDURES

RNA expression analysis and RT-qPCR

Samples were harvested in Trizol® reagent (Invitrogen). After digestion with DNase I (Invitrogen), 0.5 µg of RNA was used for cDNA synthesis. Quantitative Polymerase Chain Reactions (RT-qPCRs) were then performed using the Sybr Green gene expression assay. Real-time PCR mixtures were prepared following the manufacturer's recommendation (LightCycler® 480 SYBR Green I Master, Roche) for each gene. qPCRs were run in a Roche Lightcycler 480® and normalized to glyceraldehyde 3-phosphate dehydrogenase (GAPDH) in the same run. Data was analyzed using the $2^{-\Delta\Delta CT}$ method (Pfaffl, 2001). Statistical analysis was performed using ANOVA 1way or 2way as appropriate (see figure legends for details), followed by the Bonferroni post hoc test. Primer sequences are available in the supplemental information (**Table S2**).

Western Blot and Protein Quantification

Samples were harvested in Cellytic-M (Sigma). Protein extraction was performed following manufacturer's recommendations. Equal volumes of protein (15µl) were loaded on a gradient 4-12% Bis-Tris NuPage Gel (Life Technologies, USA) and run at 150V for 60 minutes. Wet transfer was performed on nitrocellulose membranes using NuPage transfer Buffer (Life Technologies, USA) and run at 60V for 90 minutes. Blocking and probing of the membranes was performed following manufacturer's instructions (**Table S3** for Antibodies). Membranes were washed and developed using Super Signal West Pico Chemiluminescent Substrate (Thermo Scientific, USA) and Amersham Hyperfilm ECL (Amersham, USA) as per manufacturer's instruction. Densitometric analysis was performed using Image Studio Lite Ver. 4.0 Software (LICOR Biosciences, USA) and values were normalized with loading control (β-Actin or PCNA).

Immunocytochemistry

Cell were grown on Ibi-Treat slides (Ibidi, Germany) and fixed in 4% paraformaldehyde for 10 minutes at RT. A 10 minute incubation at -20˚C in methanol allowed cell permeabilization. Following one hour of blocking in TBS/0.1% Triton-X100/5% goat serum, primary and secondary antibody staining were carried out in the blocking solution, overnight at 4˚C and for 1 hour at RT. For the list of Antibodies used, see Supplemental information (**Table S3**).

Reference:

Pfaffl, M.W. (2001). A new mathematical model for relative quantification in real-time RT-PCR. Nucleic Acids Res *29,* e45.

SUPPLEMENTAL TABLES

Table S1: Expression profile of Wnt signals, receptors and co-receptors in other model organisms

Table S2: Primers for qPCR

Table S3: Antibodies

SUPPLEMENTAL MOVIES

Movie S1: The AB+WNTi Protocol leads to development of functionally active cardiomyocytes. Movie shows beating cardiomyocytes on Day6 of the AB+WNTi Protocol.

Movie S2: The C+WNTi Protocol leads to development of functionally active cardiomyocytes. Movie shows beating cardiomyocytes on Day9 of the C+WNTi Protocol.

Table S1

GENE	RELEVANT EXPRESSION IN THE MOUSE	REFERENCES
Wnt2a	▪ Anterior lateral plate mesoderm<br>▪ Cardiac crescent (overlapping with expression of *Nkx2.5* and *Tbx5* presumably in First Heart Field)<br>▪ Foregut endoderm next to cardiac mesoderm, later restricted to pericardium and the posterior pole	(Cohen et al., 2008; Goss et al., 2009; Kemp et al., 2005; Monkley et al., 1996; Tian et al., 2010)
Wnt2b **(aka *Wnt13*)**	▪ Anterior lateral plate mesoderm<br>▪ Cardiac crescent<br>▪ Foregut endoderm next to cardiac mesoderm<br>▪ Venous pole (overlapping with expression of *Tbx18*) atrioventricular region of linear heart tube	(Goss et al., 2009; Kemp et al., 2005; Yamaguchi, 2008; Zakin et al., 1998)
Wnt3	▪ Earliest mesoderm before primitive streak formation	(Liu et al., 1999)
Wnt3a	▪ Primitive streak (overlapping with expression of *Bry*(*T*))<br>▪ Posterior embryonic mesoderm	(Takada et al., 1994; Yamaguchi, 2008)
Wnt5a	▪ Node<br>▪ Primitive streak<br>▪ Primordium of the outflow tract<br>▪ Common ventricle	(Cohen et al., 2012; Schleiffarth et al., 2007; Yamaguchi, 2008)
Wnt5b	▪ Expressed in the embryo and in the adult heart	(Gavin et al., 1990; Yamaguchi, 2008)
Wnt6	▪ Cardiac precursor cells	(Schmeckpeper et al., 2015)
Wnt8a **(aka *Wnt8*)**	▪ Primitive streak<br>▪ Tubular heart	(Jaspard et al., 2000)
Wnt9b **(aka *Wnt15*)**	▪ Epicardium<br>▪ Coronary vasculature	(Merki et al., 2005)
Wnt11	▪ Node<br>▪ Cardiac crescent (FHF/SHF?)<br>▪ Linear heart tube<br>▪ OFT myocardium<br>▪ Ventricle	(Nagy et al., 2010; Terami et al., 2004; Yamaguchi, 2008)
Fzd2	▪ Cardiac Neural Crest Cells	(van Gijn et al., 2001)
Fzd4	▪ No information on expression, but associated with human heart and great vessel patterning defects	(DeRossi et al., 2000)
Fzd5	▪ Distal, then anterior visceral endoderm<br>▪ Anterior mesendoderm	(Kemp et al., 2007)
Fzd6	▪ Notochord<br>▪ Foregut endoderm	(Borello et al., 1999)
Fzd7	▪ Wide expression domain including common cardiac progenitor	(Kemp et al., 2007; Sagara et al., 1998)
Fzd9	▪ Embryonic and adult heart and cava veins	(Wang et al., 1999)
Ror1	▪ Myocardium<br>▪ Interventricular septum<br>▪ Aortic valve and atrium, but not aortic arch or in the epicardium	(Al-Shawi et al., 2001; Matsuda et al., 2001)
Ror2	▪ Myocardium<br>▪ Interventricular septum<br>▪ Aortic valve and atrium, but not aortic arch or in the epicardium	(Al-Shawi et al., 2001; Matsuda et al., 2001)
Lrp5	▪ Embryonic heart<br>▪ Atrium and ventricle	(Visel et al., 2004)

Table S1

References:

Al-Shawi, R., Ashton, S.V., Underwood, C., and Simons, J.P. (2001). Expression of the Ror1 and Ror2 receptor tyrosine kinase genes during mouse development. Development genes and evolution *211*, 161-171.

Borello, U., Buffa, V., Sonnino, C., Melchionna, R., Vivarelli, E., and Cossu, G. (1999). Differential expression of the Wnt putative receptors Frizzled during mouse somitogenesis. Mechanisms of development *89*, 173-177.

Cohen, E.D., Miller, M.F., Wang, Z., Moon, R.T., and Morrisey, E.E. (2012). Wnt5a and Wnt11 are essential for second heart field progenitor development. Development *139*, 1931-1940.

Cohen, E.D., Tian, Y., and Morrisey, E.E. (2008). Wnt signaling: an essential regulator of cardiovascular differentiation, morphogenesis and progenitor self-renewal. Development *135*, 789-798.

DeRossi, C., Laiosa, M.D., Silverstone, A.E., and Holdener, B.C. (2000). Mouse fzd4 maps within a region of chromosome 7 important for thymus and cardiac development. Genesis *27*, 64-75.

Gavin, B.J., McMahon, J.A., and McMahon, A.P. (1990). Expression of multiple novel Wnt-1/int-1-related genes during fetal and adult mouse development. Genes & development *4*, 2319-2332.

Goss, A.M., Tian, Y., Tsukiyama, T., Cohen, E.D., Zhou, D., Lu, M.M., Yamaguchi, T.P., and Morrisey, E.E. (2009). Wnt2/2b and beta-catenin signaling are necessary and sufficient to specify lung progenitors in the foregut. Developmental cell *17*, 290-298.

Jaspard, B., Couffinhal, T., Dufourcq, P., Moreau, C., and Duplaa, C. (2000). Expression pattern of mouse sFRP-1 and mWnt-8 gene during heart morphogenesis. Mechanisms of development *90*, 263-267.

Kemp, C., Willems, E., Abdo, S., Lambiv, L., and Leyns, L. (2005). Expression of all Wnt genes and their secreted antagonists during mouse blastocyst and postimplantation development. Developmental dynamics : an official publication of the American Association of Anatomists *233*, 1064-1075.

Kemp, C.R., Willems, E., Wawrzak, D., Hendrickx, M., Agbor Agbor, T., and Leyns, L. (2007). Expression of Frizzled5, Frizzled7, and Frizzled10 during early mouse development and interactions with canonical Wnt signaling. Developmental dynamics : an official publication of the American Association of Anatomists *236*, 2011-2019.

Liu, P., Wakamiya, M., Shea, M.J., Albrecht, U., Behringer, R.R., and Bradley, A. (1999). Requirement for Wnt3 in vertebrate axis formation. Nature genetics *22*, 361-365.

Matsuda, T., Nomi, M., Ikeya, M., Kani, S., Oishi, I., Terashima, T., Takada, S., and Minami, Y. (2001). Expression of the receptor tyrosine kinase genes, Ror1 and Ror2, during mouse development. Mechanisms of development *105*, 153-156.

Merki, E., Zamora, M., Raya, A., Kawakami, Y., Wang, J., Zhang, X., Burch, J., Kubalak, S.W., Kaliman, P., Izpisua Belmonte, J.C., *et al.* (2005). Epicardial retinoid X receptor alpha is required for myocardial growth and coronary artery formation. Proceedings of the National Academy of Sciences of the United States of America *102*, 18455-18460.

Monkley, S.J., Delaney, S.J., Pennisi, D.J., Christiansen, J.H., and Wainwright, B.J. (1996). Targeted disruption of the Wnt2 gene results in placentation defects. Development *122*, 3343-3353.

Nagy, II, Railo, A., Rapila, R., Hast, T., Sormunen, R., Tavi, P., Rasanen, J., and Vainio, S.J. (2010). Wnt-11 signalling controls ventricular myocardium development by patterning N-cadherin and beta-catenin expression. Cardiovascular research *85*, 100-109.

Table S1

Sagara, N., Toda, G., Hirai, M., Terada, M., and Katoh, M. (1998). Molecular cloning, differential expression, and chromosomal localization of human frizzled-1, frizzled-2, and frizzled-7. Biochemical and biophysical research communications *252*, 117-122.

Schleiffarth, J.R., Person, A.D., Martinsen, B.J., Sukovich, D.J., Neumann, A., Baker, C.V., Lohr, J.L., Cornfield, D.N., Ekker, S.C., and Petryk, A. (2007). Wnt5a is required for cardiac outflow tract septation in mice. Pediatric research *61*, 386-391.

Schmeckpeper, J., Verma, A., Yin, L., Beigi, F., Zhang, L., Payne, A., Zhang, Z., Pratt, R.E., Dzau, V.J., and Mirotsou, M. (2015). Inhibition of Wnt6 by Sfrp2 regulates adult cardiac progenitor cell differentiation by differential modulation of Wnt pathways. Journal of molecular and cellular cardiology *85*, 215-225.

Takada, S., Stark, K.L., Shea, M.J., Vassileva, G., McMahon, J.A., and McMahon, A.P. (1994). Wnt-3a regulates somite and tailbud formation in the mouse embryo. Genes & development *8*, 174-189.

Terami, H., Hidaka, K., Katsumata, T., Iio, A., and Morisaki, T. (2004). Wnt11 facilitates embryonic stem cell differentiation to Nkx2.5-positive cardiomyocytes. Biochemical and biophysical research communications *325*, 968-975.

Tian, Y., Cohen, E.D., and Morrisey, E.E. (2010). The importance of Wnt signaling in cardiovascular development. Pediatric cardiology *31*, 342-348.

van Gijn, M.E., Blankesteijn, W.M., Smits, J.F., Hierck, B., and Gittenberger-de Groot, A.C. (2001). Frizzled 2 is transiently expressed in neural crest-containing areas during development of the heart and great arteries in the mouse. Anatomy and embryology *203*, 185-192.

Visel, A., Thaller, C., and Eichele, G. (2004). GenePaint.org: an atlas of gene expression patterns in the mouse embryo. Nucleic acids research *32*, D552-556.

Wang, Y.K., Sporle, R., Paperna, T., Schughart, K., and Francke, U. (1999). Characterization and expression pattern of the frizzled gene Fzd9, the mouse homolog of FZD9 which is deleted in Williams-Beuren syndrome. Genomics *57*, 235-248.

Yamaguchi, T.P. (2008). Genetics of Wnt signaling during early mammalian development. Methods Mol Biol *468*, 287-305.

Zakin, L.D., Mazan, S., Maury, M., Martin, N., Guenet, J.L., and Brulet, P. (1998). Structure and expression of Wnt13, a novel mouse Wnt2 related gene. Mechanisms of development *73*, 107-116.

TABLE S2: qPCR PRIMERS

Primers for RT-qPCR(Genes)	Sequences (5’-3’)
GAPDH	F: AATTCCATGGCACCGTCAAG<br>R: ATCGCCCCACTTGATTTTGG
SOX2	F: ATGACCAGCTCGCAGACCTA<br>R: GACTTGACCACCGAACCCAT
Bry (T)	F: GCTGTGACAGGTACCCAACC<br>R: CATGCAGGTGAGTTGTCAGAA
MESP1	F: TAGGGCTAGACACTTTGAG<br>R: TTTTTGCCAACTGACACC
MIXL1	F: GGTACCCCGACATCCACTT<br>R: TGGAAGGATTTCCCACTCTG
MSX1	F: GAGTTCTCCAGCTCGCTCA<br>R: CCAGCTCTGCCTCTTGTAGTC
NKX2.5	F: CAAGTGTGCGTCTGCCTTTC<br>R: CGCGCACAGCTCTTTCTTTT
TBX5	F: GGAGCTGCACAGAATGTCAA<br>R: TGCTGAAAGGACTGTGGTTG
cTnT	F: TCCCCGATGGAGAGAGAGTG<br>R: ACGAGCTCCTCCTCCTCTTT
MYH6	F: CTCAAGCTCATGGCCACTCT<br>R: GCCTCCTTTGCTTTTACCACT
ACTN2	F: CTGCTGCTTTGGTGTCAGAG<br>R: TTCCTATGGGGTCATCCTTG
AXIN2	F: GTGAGGTCCACGGAAACTGT<br>R: TGGCTGGTGCAAAGACATAG
DKK1	F: CCTTGGATGGGTATTCCAGA<br>R: CCTGAGGCACAGTCTGATGA
ALCAM	F: TAACTTGCACAGCAGAAAAC<br>R: ATGCAGTCTTTGACTTCTTC
HCN4	F: GATCCTCAGCCTCTTACGCC<br>R: AGGAGCATCATGCCGATGAG
WNT1	F: CGGCGTTTATCTTCGCTATC<br>R: TTCGATGGAACCTTCTGAGC
WNT2	F: AAAGAAGATGGGAAGCGCCA<br>R: TTCATCAGGGCTCTGGCATC
WNT2B	F: CCGAGAGTGTCAGCACCAAT<br>R: TGGACTACCCCTGCTGATGA
WNT3	F: CGCACGACTATCCTGGAC<br>R: GAGGCGCTGTCATACTTGTC
WNT3A	F: TGTTGGGCCACAGTATTCCT<br>R: GGGCATGATCTCCACGTAGT
WNT4	F: CTAGCCCCGACTTCTGTGAG<br>R: TTGGACGTCTTGTTGCATGT
WNT5A	F: GCCCAGGTTGTAATTGAAGC<br>R: TGGCACAGTTTCTTCTGTCC
WNT5B	F: CGGGAGCGAGAGAAGAACT<br>R: CGTCTGCCATCTTATACACAGC

WNT6	F: CAGCCCCTTGGTTATGGAC<br>R: AACTGGAACTGGCACTCTCG
WNT7A	F: AGAAGCAAGGCCAGTACCAC<br>R: GCCTCGTTGTTGTGCAAGTT
WNT7B	F: GCAGGAAGGTTCTAGAGG<br>R: GTTGTACTTCTCCTTCAGC
WNT8A	F: AGGCTGAGAAGTGCTACCAGA<br>R: CCATTGTTTGACCCATCACA
WNT8B	F: GAAGTACCACGCAGCACTCA<br>R: GAGATGGAGCGAAAGGTGTC
WNT9A	F: GGTGTGAAGGTGATCAAG<br>R: TGCCGTCTCATACTTGTG
WNT9B	F: AGAGGAAACAAGGACCTGCG<br>R: GCACTTACACGTGGTCCTGA
WNT10A	F: CATCTTCAGCAGAGGTTTC<br>R: CAGTGCATCCAGTTGTAAG
WNT10B	F: GATACCCACAACCGCAATTC<br>R: GGGTCTCGCTCACAGAAGTC
WNT11	F: GGCCAAGTTTTCCGATGCTC<br>R:CACCCCATGGCACTTACACT
WNT16	F: GAAACTGGATGTGGTTGG<br>R:TCATGCAGTTCCATCTCTC
FZD1	F: CATCGTCATCGCCTGCTACT<br>R: TAGCGTAGCTCTTGCAGCTC
FZD2	F: CTTCTCACAGGAGGAGACGC<br>R; AAATGATAGGCCGCTCTGGG
FZD3	F: ACAGCAAAGTGAGCAGCTACC<br>R: CTGTAACTGCAGGGCGTGTA
FZD4	F: TCAAGAGACGCTGTGAACCC<br>R: GGTCGTTCTGTGGTGGGAAT
FZD5	F: GCACAACCACATCCACTACG<br>R: GCCATGCCGAAGAAGTAGAC
FZD6	F: AGGCTTGCACCGTTTTGTTC<br>R: TGCTCGATGGCTTCACAACT
FZD7	F: CGCCTCTGTTCGTCTACCTC<br>R: TCATGATGGTGCGGATACGG
FZD8	F: CGCCTCTGTTCGTCTACCTC<br>R: TCATGATGGTGCGGATACGG
FZD9	F: TGCTCACCTTCTTGCTGGAG<br>R: GCCAGCGAGTAGACGTTGTA
FZD10	F: AAGAAGAGCCGGAGAAAACC<br>R: GACTGGGCAGGGATCTCATA
LRP5	F: CACCACCTTCTTGCTGTTCA<br>R: GCTTTGACGTTCCTCAGTCC
LRP6	F: GACTGGGTTGCACGAAATCT<br>R: CGGGGTTCCTCTAAGTCCTC
RYK	F: CCGCATACCTGAAAGATGGT<br>R: GATCTAAGGCCCAGCAACAG
ROR2	F: CCCCTCATTAACCAGCACAA<br>R: TTCCCAAACCGGTCCTCT

TABLE S3: ANTIBODIES

Primary Antibodies				
Antibody reactivity	Dilution	Application	Manufacturer	Catalogue #
OCT4	1/500	ICC	Abcam	Ab137427
Bry (T)	1/50	ICC	Abcam	Ab20680
NKX2.5	1/800	ICC	Cell Signalling Technology	8792S
MYH6	1/100	ICC	Proteintech	22281-1-AP
Active Beta Catenin	1/500<br>1/1000	ICC<br>WB	Millipore	05-665
Phospho-SAPK/JNK	1/400	ICC	Cell Signalling Technology	9255S
DAPI	1/1000	ICC	Thermo Scientific	62248
Phospho-SAPK/JNK	1/500	WB	Cell Signalling Technology	9255S
Beta Catenin	1/1000	WB	Santa Cruz	SC-7199
SAPK/JNK	1/1000	WB	Cell Signalling Technology	9251S
Beta Actin	1/1000	WB	Cell Signalling Technology	4970S

Secondary Antibodies				
Antibody reactivity	Dilution	Application	Manufacturer	Catalogue #
Goat anti-Rabbit IgG (H+L) Alexa Fluor® 488 conjugate	1/400	ICC	Thermo Scientific	A-11008
Goat anti-Mouse IgG (H+L) Alexa Fluor® 488 conjugate	1/400	ICC	Thermo Scientific	A-11001
Anti-rabbit IgG, HRP-linked	1/10000	WB	Cell Signalling Technology	7074
Anti-mouse IgG, HRP-linked	1/10000	WB	Cell Signalling Technology	7076
